# Automatic segmentation and PI-RADS grading of prostate cancer for biparametric MRI

**DOI:** 10.3389/fmed.2026.1755311

**Published:** 2026-04-13

**Authors:** Yuchun Li, Mengxing Huang, Yuanyuan Wu, Yuhan Zhang

**Affiliations:** 1 School of Information Science and Technology, Hainan Normal University, Haikou, Hainan, China; 2 State Key Laboratory of Marine Resource Utilization in South China Sea, School of Information and Communication Engineering, Hainan University, Haikou, Hainan, China; 3 School of Electronic and Information Engineering, Guangdong Ocean University, Zhanjiang, China; 4 School of Biomedical Engineering, Medical School, Shenzhen University, Shenzhen, Guangdong, China

**Keywords:** biparametric MRI, image fusion, multitask learning, PI-RADS grading, prostatic cancer

## Abstract

The annual mortality rate from prostate cancer (PCa), a common malignant neoplasm affecting middle-aged and elderly men, is on the rise. Biparametric magnetic resonance imaging (bpMRI) is indispensable to PCa imaging analysis since it can capture distinct disease-related information from two modalities that exhibit synergistic performance. The majority of state-of-the-art PCa diagnostic techniques currently available are focus on a single modality or task, neglecting the information sharing across the two modalities and task correlations inherent in multi-task learning. We provide a dual-modality image fusion and multi-task learning model that can accomplish both automatic PI-RADS grading and prostate and PCa region segmentation simultaneously. First, to extract complementary information between the prostate and PCa in bimodal images via T2-weighted imaging (T2WI) and diffusion-weighted imaging (DWI) feature extraction, a shared block fusion module and an independent encoder block were developed; Subsequently, in the encoder stage, the dual visual attention module was designed to extract features from multiple receptive field and deliver more accurate contextual information, and a novel decoder was designed to effectively integrate encoder features, yielding more refined global and local detail information; Next, to capture more precise detail information during the classification task stage, a high-level feature fusion technique was developed; To address class imbalance, a multitask mixed loss function is finally suggested. The segmentation results of prostate and PCa on multiple diverse male pelvic MRI datasets demonstrate the superior performance of our proposed method. Both the basic performance evaluation and comparative model evaluation of the proposed model have validated its effectiveness in prostate and PCa segmentation as well as PI-RADS automatic grading. External validation on the independent PROMISE12 dataset further confirms the strong generalizability of our model across different institutions, scanning devices and patient cohorts.

## 1 Introduction

Prostate Cancer (PCa) is the second leading cause of cancer-related mortality for men globally and is a common malignant neoplasm in middle-aged and elderly men. According to statistical data, 1.41 million instances of PCa were diagnosed annually in 2022, accounting for 14.1% of all cases of cancer in males (1). Due to its ability to provide multiparametric, high-contrast, and multiplanar imaging, magnetic resonance imaging (MRI) is a widely used diagnostic tool for PCa detection and segmentation (2). MRI can visualize the integrity of the prostate capsule, the invasion of adjacent tissues by PCa, and whether there are any metastases to the bone or pelvic lymph nodes. In clinical practice, pre-biopsy MRI for high-risk male patients has rapidly become the clinical standard of care (3). The Prostate Imaging Reporting and Data System (PI-RADS), a consensus guideline developed by radiologists, was established to describe and report PCa. While PI-RADS version 2 recommends the use of dynamic contrast-enhanced (DCE) sequences, diffusion-weighted imaging (DWI), and T2-weighted imaging (T2WI) to locate and detect PCa, DCE imaging is only marginally significant when DWI suspects peripheral zone PCa clinical significance. Prior to PI-RADS version 2 was established, a number of studies have demonstrated that, when compared to mpMRI, biparametric magnetic resonance imaging (bpMRI) which only incorporates T2WI and DWI has comparable diagnostic accuracy. Additionally, bpMRI acquisition takes only 17 min, compared with mpMRI acquisition takes 45 min. The need for clinicians to monitor adverse reactions to the intravenous contrast agents used in DCE imaging. bpMRI is a powerful alternative for mpMRI, offering significant reductions in imaging time and cost (4).

**Figure 1:**
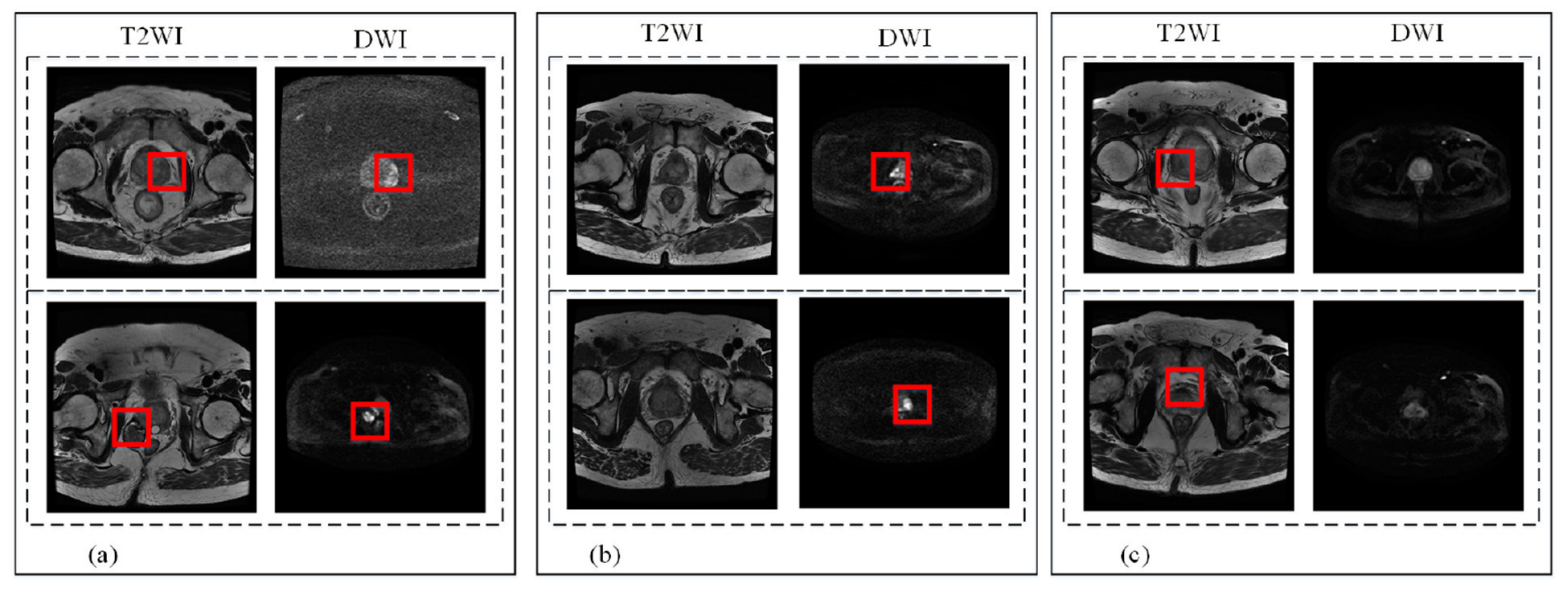
Imaging manifestations of prostate cancer (PCa) in dual-modal bpMRI. **(a)** Cancer appears in both modes. **(b)** Manifestation in cancer in DWI. **(c)** Manifestation in cancer T2WI.

The PI-RADS grading system, together with standardized mpMRI scanning, analysis, and reporting protocols, provides a comprehensive set of guidelines for mpMRI-based PCa assessment (5). Combining PI-RADS grading with mpMRI, PCa can be diagnosed and risk stratification, providing better guidance for a subsequent targeted biopsy and significantly reducing the rate of overdiagnosis (6). T2WI is based on the spin motion of water molecules in tissues. T2WI imaging is characterized by relatively few artifacts, a shorter scanning time, and a clear visualization of PCa. T2WI imaging can provide a detailed anatomical map of the prostate, which aids clinicians in accurately localizing PCa and making clinical decisions. DWI is a technique that quantifies the diffusion of water molecules to evaluate the cellular and tissue microstructure of the prostate (7). The human body's water molecules, both bound and free, random Brownian motion, and is the fundamental physical basis of DWI. The diagnostic value of DWI for PCa stems from the significant differences in diffusion coefficient values between PCa tissue and normal prostate tissue. Approximately 70% of peripheral zone PCa can be detected by DWI, and DWI is the primary sequence with advantages in transition zone PCa detection (8). Each imaging modality has benefits of its own and is necessary for the diagnosis of PCa. However, the sole use of T2WI or DWI for PCa identification, T2WI imaging and DWI may lead to misdiagnosis. Figure 1 displays the imaging manifestations of PCa on T2WI and DWI, with the cancerous region marked in the color box Figure 1a shows cancer appears in both modes, while Figure 1b shows manifestations of cancer in DWI, which is difficult to distinguish on T2WI. Figure 1c shows manifestations of cancer in T2WI, which not clearly visualized in DWI mode. Therefore, the synergistic application of T2WI and DWI in dual-modality bpMRI can improve the performance of PCa segmentation. Currently, clinicians manually delineate of tissue regions on 3D MRI slices to assessment PCa. However, this task is time-consuming and labor-intensive, increases the workload of radiologists. The majority of studies on prostate MRI image segmentation use T2WI due to its clear depiction of the internal anatomical structure of the prostate gland. Many studies have been conducted on prostate region segmentation of the prostate area (9–12). Over the past two years, researchers have focused increasingly on PCa detection and segmentation (13–17). Additional studies have explored multimodal fusion of PCa detection and segmentation (18) and PCa detection and segmentation on DWI images alone (19). Existing methods typically handle PCa volume quantification as a standard semantic segmentation task, ignoring clinical practice requirements. State-of-the-art multimodal transformer-based segmentation methods such as 3D TransUNet (20), DCTNet (21), and TMA-TransBTS (22) mainly focus on single-task segmentation for general organs or specific types of PCa, and lack the integration of clinical grading tasks and targeted optimization for PCa bpMRI. These methods adopt simple channel concatenation, separate feature extraction with late fusion, or mid-stage cross-attention fusion based on pre-aligned features, which fail to fully exploit the complementary information between T2WI and DWI for PCa diagnosis the complementary information between T2WI and DWI in PCa diagnosis. In clinical practice, radiologists often localize specific PCa foci by analyzing multimodal imaging data. PCa is influenced by the extent and location of malignant transformation; on T2WI and DWI, the imaging manifestations of PCa on T2WI and DWI differ significantly in terms of size, shape, and ill-defined boundaries. Therefore, it is crucial to develop a bpMRI-based method for prostate and PCa segmentation and detection. Currently, most researchers focus on the segmentation task of the prostate or PCa and and few studies have addressed the automatic PI-RADS grading of PCa. Therefore, the integration of the PI-RADS classification task and PCa segmentation task in a unified learning framework is expected to, improve the performance of both PCa segmentation and grading.

The most widely used method for classifying or segmenting multimodal medical images is to use multiple encoder branches to individually extract information from each modality. When employing this method, T2WI and DWI are unable to support one another during the feature extraction process. However, given the subject on the T2WI and DWI scans is the same, the PCa should appear in the same location on both scans. For example, during learning, both scanning modalities should apply exact labels and PCa should be situated in a steady spatial location. In cases when the anatomical structure of DWI images is not obvious, shortcomings can be corrected by adjusting and attenuating T2WI images. Meanwhile, the T2WI qualitative and quantitative analysis reflects the functioning characteristics of the organs.

We have already developed an interactive automatic labeling method (23) for the prostate and a single-mode automatic segmentation model (24) for the prostate and PCa in the early stage. Therefore, we propose a PCa automatic segmentation and PI-RADS automatic scoring model based on multi task learning. Related tasks can share complementary information, thereby improving model performance. To segregate the prostate and PCa regions and automatically grade the PI-RADS, this paper suggests using multimodal image fusion and multitask learning neural networks. Transformer technology was added at the end of the encoder, visual attention method and shared block fusion downsampling method were established in the encoder, and a high-level feature fusion module was designed in the classification task. We also constructed a mixed loss function for segmentation and classification to handle class imbalance. The contributions of this study are as follows:

(1) A novel dual-mode fusion 3D multi task learning network structure is proposed for automatic segmentation of prostate and PCa on bpMRI and PI-RADS automatic grading, which is the first method to integrate PCa segmentation and PI-RADS grading into a unified framework among the latest multimodal transformer-based segmentation approaches, aligning with the clinical workflow of “one-stop” PCa diagnosis.

(2) A dual visual attention mechanism was designed in the encoder stage to capture features from different receptive fields by combining standard convolution and multi-dilation-rate dilated convolution (1/3/5/7), avoiding the gridding effect of single dilated convolution and providing more comprehensive multi-scale feature information without increasing computational complexity.

(3) A new decoder was designed during the segmentation task phase to effectively integrate encoder functionality, obtaining more refined PCa global and local detail information. A high-level feature fusion (HLFF) module was designed during the classification task phase to capture the clinical PI-RADS scoring criteria by fusing multi-scale features before and after the Transformer execution.

(4) A multi task mixed loss function with adaptive weights was constructed to handle class imbalance, which solves the problem of class differences in prostate, PCa, and background regions in segmentation tasks and dynamically balances the segmentation and grading tasks during training.

(5) The algorithm was evaluated on PCa bpMRI images obtained from a local hospital (98 patients) and an independent external validation dataset (PROMISE12, 50 cases). Comprehensive comparative experiments with classical CNN baselines and recent SOTA multimodal transformer methods and rigorous statistical analysis (paired *t*-tests, Wilcoxon rank-sum tests, 95% confidence intervals) verify the novelty and effectiveness of the proposed method.

## 2 Methods

### 2.1 Overview of dual-modal multitask learning model

**Figure 2:**
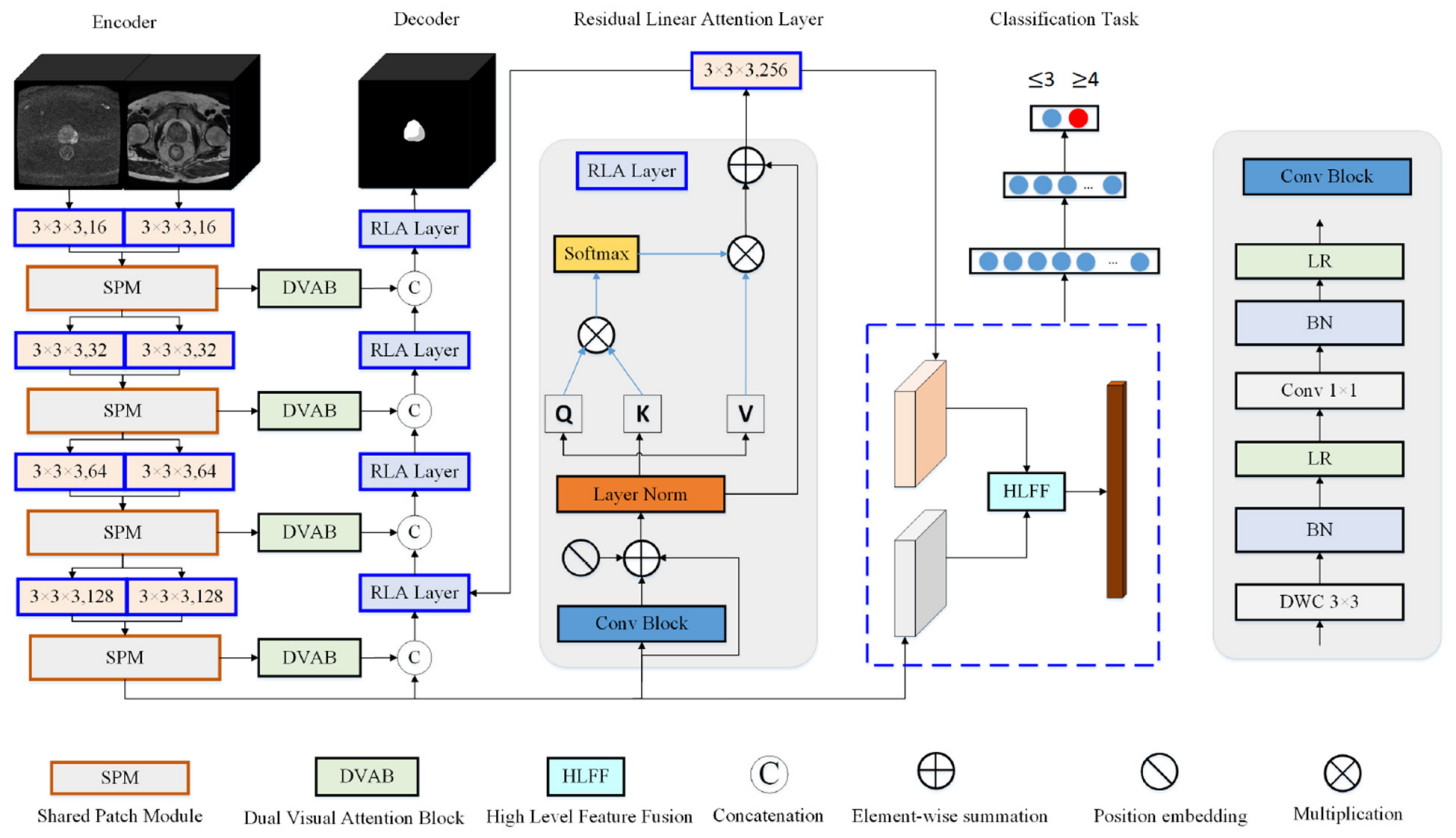
Framework of the proposed multimodal and multitasking 3D network.

The dual-mode multitask 3D automatic segmentation and PI-RADS scoring model for the prostate and PCa presented in this work is depicted in Figure 2 as its structural diagram. The robust, universal, end-to-end V-Net (25) serves as the foundation for the model structure. There are four components to the structural diagram: (1) the context-encoding encoder section; (2) residual linear attention layer for global representation learning; (3) the prostate and PCa segmentation decoder section; and (4) classifier for PI-RADS automatic grading. This model uses 3D images of T2WI and DWI from two modalities of mpMRI as inputs. SPM mechanism was designed in the encoder section to downsample features and promote feature sharing. This part of the structure achieves bimodal image fusion, and the feature interaction process is shown in Figure 2. The features extracted by the residual linear attention module are used for both segmentation tasks and PI-RADS grading tasks. Moreover, a dual visual attention mechanism has been added to the decoder part of the model to achieve the segmentation task of prostate and PCa. The high level feature fusion (HLFF) module extracts finer features for implementing PI-RADS grading tasks. Dual modal image fusion provides a rich feature foundation for multi task learning, and the effective completion of each task relies on different components to provide remote contextual modeling and more refined high-level features. Firstly, define the following symbols: ${\text{D}}_{\text{L}}={\left\{{\text{x}}^{\left(\text{i}\right)},{\text{y}}^{\left(\text{i}\right)},{\text{g}}^{\left(\text{i}\right)}\right\}}_{\text{i}=1}^{{\text{S}}_{\text{L}}}$ represents the label set of sample S_L_, where x^(i)^ represents input bimodal (T2WI + DWI) 3D bpMRI volume of the *i*−*th* sample; $\text{x}\in {ℛ}^{\text{W}\times \text{H}\times \text{D}\times \text{C}}$ (*W*: width, *H*: height, *D*: depth, *C*: number of channels), and y^(i)^ represents the ground-truth label map of the *i*−*th* sample for prostate/PCa segmentation (pixel-wise binary label: 1 = target region [prostate/PCa], 0 = background). ${\text{g}}^{\left(\text{i}\right)}={\left\{{\text{g}}^{\left(\text{i}\right)}|{\text{g}}^{\left(\text{i}\right)}\in \left\{0,1\right\}\right\}}_{\text{i}=1}^{{\text{S}}_{\text{L}}}$ represents a PI-RADS score of ≤ 3 or ≥4. For the i-th sample, g^(i)^ = 0 indicates a PI-RADS score of ≤ 3, and g^(i)^ = 1 indicates a PI-RADS score of ≥4. To capture remote dependencies restricted by CNN, a residual linear attention layer is introduced at the decoder to achieve efficient remote context modeling.

Two 3 × 3 × 3 convolutional layers make up the encoder stage's input, which is then processed by batch normalization (BN) and parameter rectification linear unit (PReLU) activation. Dual visual attention was intended to extract features of various scales in the encoder stage. These characteristics were then applied to attention to acquire feature information with various receptive fields. To preserve structural and spatial consistency, a shared block fusion method was proposed for the encoder's down-sampling stage. This mechanism maps T2WI and DWI pictures to the same geographic region of interest. Incorporate residual linear attention layer at the decoder to improve the way features are represented and to learn more about global features. To gather more precise data, a HLFF method was built prior to the PI-RADS automatic classification classifier. Segmentation probability maps for the prostate and PCa are obtained by the decoder stage by integrating residual linear attention layer features and dual visual attention features. The segmentation job step produces an identically sized end-to-end probability map for every 3D input block. We shall go into great length about the suggested model and its details on multimodal and multitask shared learning in the sections that follow. The model can perform PI-RADS automatic grading tasks and segment prostate and PCa on multimodal MRI images with complicated backgrounds.

### 2.2 Encoder module

**Figure 3:**
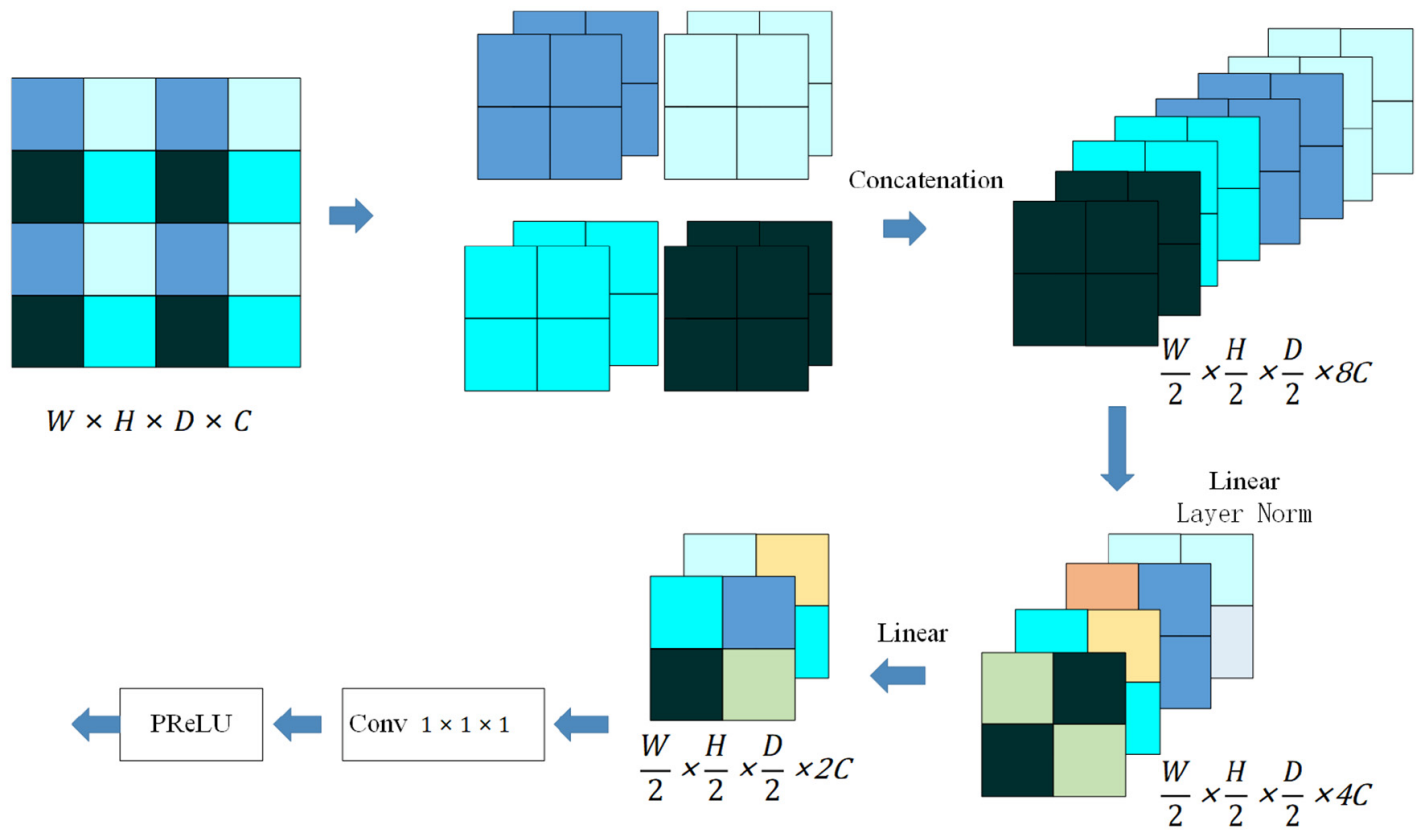
The proposed Shared Patch Module (SPM) structure.

The encoder uses a series of convolution and transformation operations to extract global semantic features from the input $\text{x}\in {ℛ}^{\text{W}\times \text{H}\times \text{D}\times \text{C}}$ (3D spatial resolution is W × H × D, *C* indicates the number of channels). Firstly, perform an initial convolution operation on the input kernel size to generate a feature map with 16 channels. In order to capture spatial and depth feature representations, a feature map $\text{F}\in {ℛ}^{{\text{W}}^{\prime }\times {\text{H}}^{\prime }\times {\text{D}}^{\prime }\times {\text{C}}^{\prime }}$ is generated through SPM and multiple stacked convolutional blocks. Each convolutional block consists of batch normalization, Leaky ReLU function, and convolution. When multimodal medical images are used for segmentation or detection tasks, the most common method is to use multiple encoder branches to extract features from each modality separately. Using this method, T2WI and DWI cannot benefit each other during the feature extraction process. On this basis, an SPM module was designed to assist the encoder branch and improve the effectiveness of feature extraction. The theoretical innovation of SPM compared with existing fusion mechanisms is to address the “modal disconnect” in traditional dual-branch encoders by enforcing spatial consistency of multi-modal features. During the training process, the weights of SPM are updated twice within one training cycle (the first input of SPM is the feature encoding of T2WI, and the second input is the feature encoding of DWI), enabling the model to learn cross-modal complementary patterns (e.g., T2WI's anatomical context and DWI's diffusion characteristics). Using the SPM module to map T2WI and DWI feature maps to the same spatial region of interest can maintain spatial and structural consistency. As shown in Figure 3, assuming that the input SPM is a feature map with a 3D spatial resolution of W × H × D and a number of channels of *C*, block fusion will divide each adjacent pixel of (W/2) × (H/2) × (D/2) into a block, and then concatenate each pixel at the same position to obtain 8 feature maps. Next, concatenate these eight feature maps, and then use a regularization layer and two fully connected layers to linearly change the number of channels in the feature map from *C* to (C/2). After the block fusion operation, the feature size will be halved and the number of channels will double. In addition, 1 × 1 × 1 convolution promotes information exchange between T2WI and DWI channels, improving the non-linear expression of the model. The encoder branch has independent encoder blocks and the same SPM for feature extraction for T2WI and DWI. During the training process, the weights of SPM are updated twice within one training cycle (the first input of SPM is the feature encoding of T2WI, and the second input is the feature encoding of DWI). Assuming that when the input of SPM comes from T2WI, the output of SPM can contain some corresponding DWI information, and vice versa. This also means that the output of each SPM can represent information from two modalities, namely T2WI and DWI. This module allows T2WI to identify regions of PCa that are not readily visible in signal T2WI, as well as eliminate false positives from DWI feature maps. However, T2WI features help to distinguish PCa areas with similar anatomical features to surrounding organs. This module allows T2WI to identify regions of PCa that are not readily visible in DWI, as well as eliminate false positives from DWI feature maps, and DWI features help to distinguish PCa areas with similar anatomical features to surrounding organs in T2WI.

### 2.3 Dual visual attention mechanism

**Figure 4:**
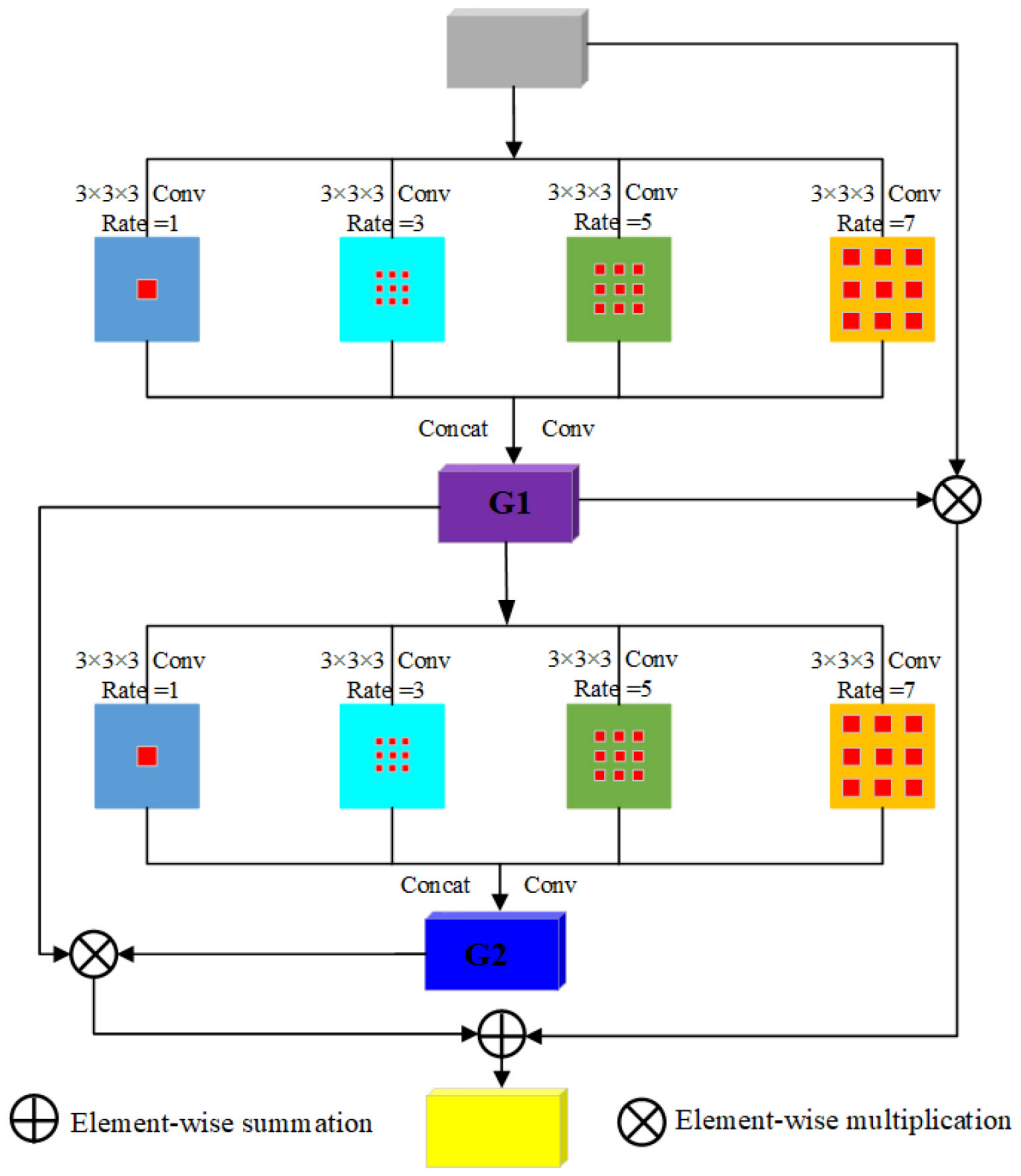
The proposed dual visual attention mechanism.

Prostate and PCa segmentation requires pixel level classification of images, which not only accurately identifies the categories of different objects in the image, but also determines the specific location of each object in the image. This requires the model to have a large receptive field to capture information from a wider area, while not increasing computational costs and parameter count due to excessive convolution layers. Therefore, our dual visual attention mechanism adopts dilated convolution. Expanding convolution can significantly increase the receptive field without increasing the size of the convolution kernel or the number of convolution layers. It achieves this effect by inserting dilation rates between convolutional kernel elements. However, as the number of dilated convolution layers increases, the number of parameters also gradually increases. Therefore, to ensure that sufficient multi-scale information can be obtained while maintaining the efficiency of the model, we choose to use two dilated convolution groups to implement the attention mechanism. The theoretical innovation of the dual visual attention mechanism compared with existing attention designs is to combine standard convolution and multi-dilation-rate dilated convolution (1/3/5/7) to avoid the 'gridding effect' of single dilated convolution, and achieve efficient multi-scale feature aggregation without increasing computational complexity. Figure 4 shows the attention structure diagram in the 3D segmentation network of the proposed method for prostate and cancerous tissue. The input scale of the model is the feature map F_i_(i = 1, 2, 3, 4) and the output is an attention feature ${\text{F}}_{\text{i}}^{\text{a}}$ of the same size as F_i_. In the designed dual visual attention module, dilated convolutional layers with different dilation rates increase the visual receptive field. The inspiration for the attention module comes from the aim of obtaining global and local information through dilated convolution. Attention contains two layers of dilated convolutional groups with different dilation rates. In our proposed attention, each set of dilated convolutional blocks is used to expand the receptive field, with dilation rates of 1, 3, 5, and 7, respectively. The input feature F_i_ is subjected to dilation convolutions with dilation rates of 1, 3, 5, and 7 in sequence. Then, the results of each dilation convolution are concatenated and convolved. The feature map G1 obtained from the first dilation convolution is input to the lower two dilation convolution groups, and dilation convolutions with dilation rates of 1, 3, 5, and 7 are performed in sequence. Then, the results of each dilation convolution are concatenated to output the feature map G2. Finally, F_i_ is multiplied by G1 element by element, and the results of G1 and G2 multiplied element by element are added up element by element to obtain the final feature. The feature map generated by fusion and activated by the sigmoid function is multiplied and added with the input and intermediate features to obtain the final attention feature ${\text{F}}_{\text{i}}^{\text{a}}$. Different from the attention mechanisms of 3D TransUNet (global self-attention ignores local details), DCTNet (lacks optimization for small PCa foci) and TMA-TransBTS (insufficient integration of local and global features), our dual visual attention mechanism can simultaneously capture the local fine features (e.g., PCa boundaries) and global contextual features (e.g., prostate-PCa spatial relationships) of PCa. Moreover, the attention mechanism serves both segmentation (pixel-wise classification) and grading (semantic feature extraction) tasks, while existing attention mechanisms are solely designed for segmentation tasks.

### 2.4 Residual linear attention layer

We created a residual linear attention layer in order to efficiently integrate and enhance the features between the encoder's skip connections and the decoder's feature maps. Each layer's dual visual attention mechanism output features are cascaded with the residual linear attention layer and fed into the subsequent residual linear attention layer during the decoder stage. This cascade promotes the comprehensive feature representation that makes use of data from both the encoder and decoder routes by fusing the context-rich low resolution features of the decoder with the high-resolution features of the encoder. After a convolution block refines the initial input feature, thick layers are used to project the feature map onto keys, queries, and values. The softmax function is used to normalize the attention weights of keys and queries after they have been determined using the dot product.according to Equation 1. The network can prioritize the most pertinent information by using these weights to determine how much emphasis should be placed on various feature map sections. In order to retain the decoder's features while enhancing them using attention techniques, the weighted values that are acquired are then added to the original feature map through residual connections.


$$\begin{array}{l} Attn=softmax\left(\frac{QK^{T}}{\sqrt{d_{k}}}\right)V \end{array}$$ (1)

where *Attn* is the attention output (residual linear attention layer), and Q, K, and V are the projection matrices of the query matrix, key matrix, and value matrix (residual linear attention layer), respectively. The residual linear attention layer's sequence of actions functions as an attention mechanism, which is very useful for carrying out accurate feature integration and alignment at various network layers.

### 2.5 PI-RADS grading task

**Figure 5:**
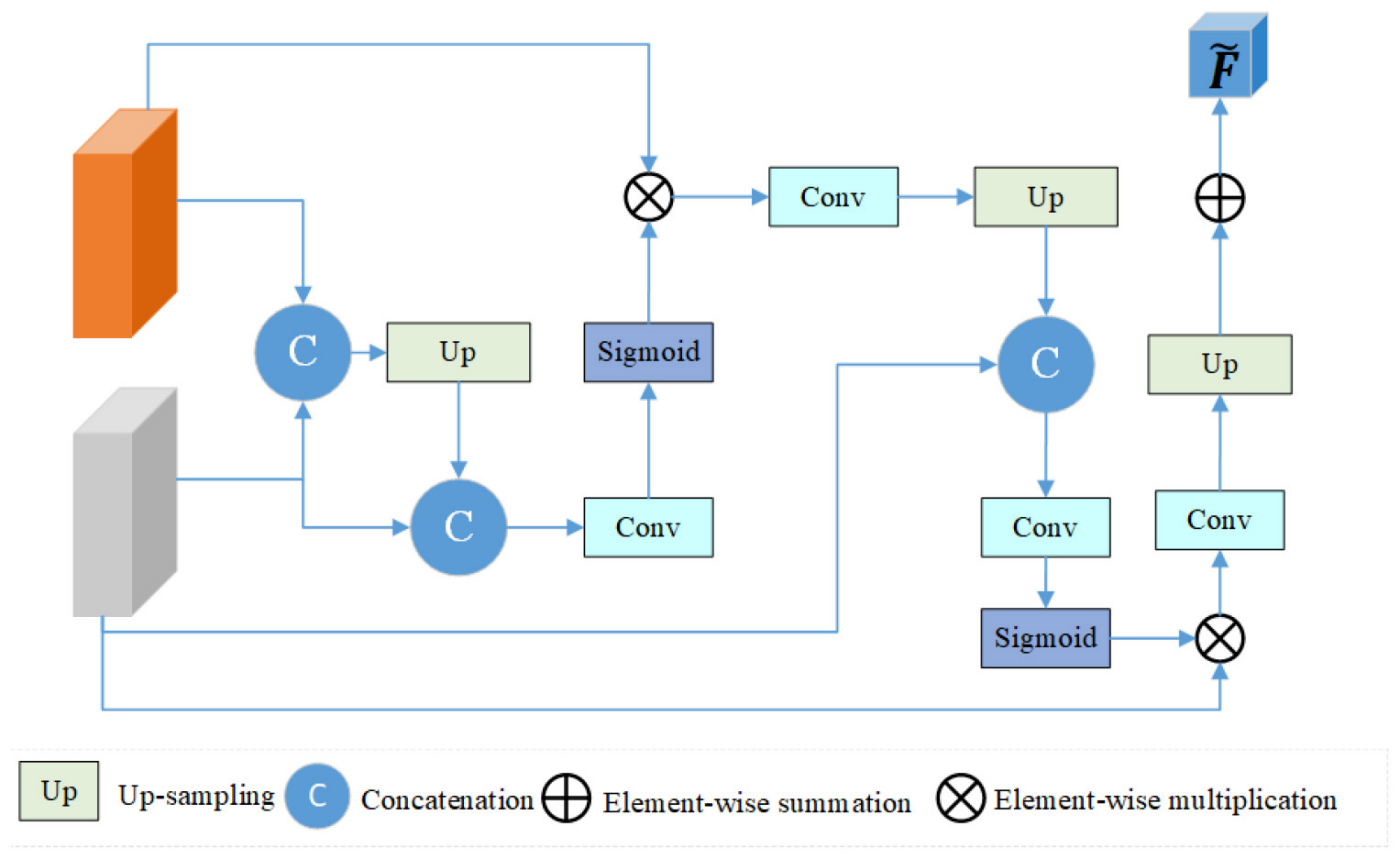
The proposed High-Level Feature Fusion (HLFF) module.

High-level features summarize target qualities, which are often utilized features in classification tasks, while low-level features primarily capture shape and boundary information. Therefore, to build a multi-scale network for PI-RADS automatic classification, high-level features at various levels both before and after the Transformer's execution are used as multi-scale features. The HLFF structure diagram in the prostate PI-RADS automatic grading 3D network of our suggested approach is displayed in Figure 5. The final high-level fused feature map, $\tilde{F}$, is produced using this structure by inputting various features both before and after the Transformer, with the features before the Transformer execution represented by *F*_1_ and the features after the Transformer execution by *F*_2_. We propose the HLFF feature learning module to better exploit the refined multi-scale features gained by various modules generated by feature learning. Rather than anticipating the refined features directly, this module first inputs the various features both before and after the Transformer execution, and then progressively combines the two features. This has the benefit of producing a predicted feature map that is more accurate. More specifically, the module converts the high-level semantic information of small-scale features into large-scale features by concatenating the features between two scales from top to bottom. Next, concatenate feature *F*_2_ from top to bottom once more after performing an up-sampling procedure. One by one, the acquired features are convolved before being entered into the sigmoid function. After multiplying this vector by the input feature *F*_1_, the resultant feature is subjected to convolution and upsampling. After that, the resulting feature is convolved and sent into the sigmoid function after being concatenated with feature *F*_2_ from top to bottom. After multiplying this vector by the input feature B, the acquired feature is subjected to a convolution sum and an upsampling operation, and the fused feature $\tilde{F}$ is output. The feature $\tilde{F}$ output by HLFF is fed as an input feature into three fully connected layers consisting of 512, 32, and 2 neurons. Finally, the Softmax function will output the probability of PI-RADS automatic classification. Our dataset roughly divides PI-RADS scores into two categories based on the probability of having prostate cancer being less than 50% and greater than or equal to 50%. Among them, PI-RADS scores ≤ 3 are classified into one category, and scores ≥4 are classified into another category. ${\text{g}}^{\left(\text{i}\right)}={\left\{{\text{g}}^{\left(\text{i}\right)}|{\text{g}}^{\left(\text{i}\right)}\in \left\{0,1\right\}\right\}}_{\text{i}=1}^{{\text{S}}_{\text{L}}}$ represents a PI-RADS score of ≤ 3 or ≥4. For the i-th sample, g^(i)^ = 0 indicates a PI-RADS score of ≤ 3, and g^(i)^ = 1 indicates a PI-RADS score of ≥4. The implementation process of HLFF can be expressed as Equations 2 and 3.


$$\begin{array}{l} \tilde{F}=up\left({\mathrm{sigmoid}}_{\left(up\left(F^{\prime }\right)⊕F_{2}\right)}⊗F_{2}\right) \end{array}$$ (2)

$$\begin{array}{l} F^{\prime }=F_{1}⊗\left({\mathrm{sigmoid}}_{\left(up\left(F_{1}⊕F_{2}\right)+F_{2}\right)}\right) \end{array}$$ (3)

where $\tilde{F}$ represents final high-level fused feature map (HLFF module for PI-RADS grading),*F*_1_ and *F*_2_ represent the two feature maps of the input, *F*′ represents the intermediate feature obtained by HLFF, *up*() represents upsampling. ⊕ represents element-wise summation (HLFF module), ⊗ represents element-wise multiplication (HLFF module and attention mechanisms).

### 2.6 Multi-task learning loss function

The theoretical innovation of our multi-task learning framework propose a “spatial-semantic mutual promotion” mechanism: segmentation provides precise PCa location/volume information to constrain grading (avoiding grading errors caused by mislocalization), and grading provides clinical semantic constraints to guide segmentation (prioritizing clinically significant lesions). Different from the single-task losses of existing methods (e.g., 3D TransUNet's Hungarian matching loss, TMA-TransBTS's multi-scale Dice loss), our multi-task mixed loss with adaptive weights dynamically balances the segmentation and grading tasks, and the optimal parameter settings of φ=0.2, γ=1, η_*D*_=0.9 and η_*F*_=0.1 are determined through extensive parameter ablation experiments. A mixed loss function, which consists of the weighted sum of the two functions, was created for the model to handle the class imbalance problem in the prostate region, PCa region, and background region in the segmentation task of multimodal multitasking learning networks. Equation 4 defines the Dice loss, which is the first loss function that is specifically intended to optimize segmentation performance assessment indicators. Equation 5 defines the second loss function, known as focal loss, which is enhanced by log loss to address the issue of unbalanced positive and negative samples. The loss function used in our method is shown in Equation 6. As a result, the segmentation task's loss function is written as follows:


$$\begin{array}{l} {ℒ}_{\text{DCE}(\text{X})}=\frac{\sum_{i=1}^{n}\left|p\left(x_{i}\right)\cap g\left(x_{i}\right)\right|}{\sum_{i=1}^{n}\frac{1}{2}\left(\left|p\left(x_{i}\right)\right|+\left|g\left(x_{i}\right)\right|\right)}=\frac{\sum_{i=1}^{n}2\left|p\left(x_{i}\right)\cap g\left(x_{i}\right)\right|}{\sum_{i=1}^{n}\left(\left|p\left(x_{i}\right)\right|+\left|g\left(x_{i}\right)\right|\right)} \end{array}$$ (4)

$$\begin{array}{l} {ℒ}_{\text{Focal}\left(X\right)}=-\frac{1}{n}\overset{n}{\underset{i=1}{\sum }}\left[\begin{array}{c} \varphi g\left(x_{i}\right){\left(1-p\left(x_{i}\right)\right)}^{\lambda }\log p\left(x_{i}\right)\\ +\\ \left(1-\varphi \right)\left(1-g\left(x_{i}\right)\right)p{\left(x_{i}\right)}^{\lambda }\\ \cdot \\ \log\left(1-p\left(x_{i}\right)\right) \end{array}\right] \end{array}$$ (5)

$$\begin{array}{l} {ℒ}_{\text{seg }}=\eta_{\text{D}}\cdot {ℒ}_{\text{DCE}(\text{X})}+\eta_{\text{F}}\cdot {ℒ}_{\text{Focal}(\text{X})} \end{array}$$ (6)

where η_D_ and η_F_ represent the weight factors of DCE loss and Focal loss, respectively, set to 0.8 and 0.2. *p*(*x*_*i*_) represents predicted probability (or predicted region) of the *i*−*th* pixel/voxel. *g*(*x*_*i*_) represents the ground-truth label of the *i*−*th* pixel/voxel (segmentation: 1 = target, 0 = background; grading: 1 = PI-RADS ≥4, 0 = PI-RADS ≤ 3). *n* represents the total number of pixels/voxels in the input volume (for segmentation metrics) or total number of samples (for loss functions). We use the most commonly used cross entropy loss function in image classification tasks as the loss function for the prostate PI-RADS automatic scoring classification task, defined as Equation 7:


$$\begin{array}{l} {ℒ}_{\text{cls}}=-\frac{1}{\;\text{K}}\sum_{\text{k}=1}^{\text{K}}\left({\text{g}}_{\text{k}}\log\left({\text{p}}_{\text{k}}\right)+\left(1-{\text{g}}_{\text{k}}\right)\log\left(1-{\text{p}}_{\text{k}}\right)\right) \end{array}$$ (7)

where, K represents the number of datasets (for PI-RADS grading cross-entropy loss), g_k_ represents the truth labels, and p_k_ represents the probability of the classification model output. Finally, the loss function of our proposed front row and PCa segmentation and PI-RADS automatic grading model based on multimodal and multi-task learning is defined as Equation 8:


$$\begin{array}{l} {ℒ}_{\text{MMTL}}=\frac{1}{2\alpha_{\text{seg}}^{2}}\cdot {ℒ}_{\text{seg}}+\frac{1}{2\alpha_{\text{cls}}^{2}}\cdot {ℒ}_{\text{cls}}+\log\alpha_{\text{seg}}\alpha_{\text{cls}} \end{array}$$ (8)

where α_seg_ and α_cls_ epresent uncertain weights and are obtained through network learning. Firstly, initialize α_seg_ and α_cls_ as two tensors with a value of 1, and then update them adaptively through iteration during the training phase.

### 2.7 Comparison with state-of-the-art multimodal transformer architectures

#### 2.7.1 Core design philosophy

Our model focuses on clinical task synergy for prostate cancer diagnosis, integrating prostate/PCa segmentation (spatial localization) and PI-RADS grading (clinical significance assessment) into a unified multitask framework. The design is tailored to the clinical workflow of biparametric MRI (bpMRI), addressing the practical need for one-stop diagnosis. 3D TransUNet (2023) is a general-purpose segmentation framework based on nnU-Net, designed for multi-organ/PCa segmentation (e.g., pancreatic PCa, hepatic vessels) without clinical grading functionality. It adopts a single-task optimization philosophy, lacking adaptation to specific disease diagnosis scenarios. DCTNet (2024) targets breast cancer lesion segmentation using dual-modal (FFOCT+DCI) data, emphasizing modality-specific feature separation and fusion to minimize cross-modal interference. It is also a single-task segmentation model, not designed for integrated clinical assessment. TMA-TransBTS (2025) specialized for brain tumor subregion segmentation (WT/ET/TC) with a focus on multi-scale PCa capture to handle heterogeneous brain tumor sizes. It is a single-task model limited to brain tumor segmentation, with no extension to clinical grading.

#### 2.7.2 Modality fusion strategy

Our model proposes an early fine-grained collaborative fusion via the Shared Patch Module (SPM). T2WI and DWI features are mapped to the same spatial region of interest (ROI) in the encoder stage, with weight sharing during training (SPM weights are updated twice per epoch to adapt to both modalities). This enables cross-modal mutual correction (e.g., using T2WI's anatomical structure to reduce DWI's false positives) and avoids the independent feature extraction limitation of traditional dual-branch encoders. The SPM is specifically optimized for the inherent modality differences of bpMRI (e.g., T2WI's 512 × 512 × 22 vs. DWI's 256 × 256 × 22 voxel size) to adaptively align spatial resolutions. 3D TransUNet (2023) lacks a dedicated multimodal fusion module. Multi-modal data are simply concatenated as additional input channels, with no interaction between modalities during feature extraction. Fusion is superficial and not optimized for cross-modal complementary information. DCTNet (2024) uses a two-branch separation-fusion strategy: dual CNN encoders extract modality-specific features, and a Transformer encoder extracts cross-modal shared features, followed by late fusion via the Cross-Modal Feature Fusion Module (CFM). This design leads to feature redundancy and fails to leverage early cross-modal synergy. TMA-TransBTS (2025) adopts mid-stage cross-attention fusion via the 3D Multi-scale Crossattention Module (TMCM), which replaces traditional skip connections in the decoder to fuse encoder multi-scale features with decoder features. However, it relies on pre-aligned multi-modal features and does not address spatial/resolution differences between modalities (e.g., DWI vs. T2WI in bpMRI).

#### 2.7.3 Attention mechanism

Our model proposes a dual visual attention mechanism that combines standard convolution (for local fine features, e.g., PCa boundaries) and multi-dilation-rate dilated convolution (1/3/5/7) (for global contextual features, e.g., prostate-PCa spatial relationships). This mechanism avoids the gridding effect of single dilated convolution and achieves efficient multi-scale feature aggregation without increasing computational complexity. The attention mechanism serves both segmentation (pixel-wise classification) and grading (semantic feature extraction), realizing task synergy of feature extraction. 3D TransUNet (2023) adopts global self-attention+query cross-attention. The encoder's global self-attention tokenizes CNN features to model long-range dependencies but ignores local detail preservation; the decoder's query-based attention is designed for mask classification, not optimized for multi-scale medical image features of PCa. DCTNet (2024) uses cross-attention + Conformer, focusing on modality-specific feature interaction but lacking targeted optimization for small, irregular lesions (e.g., early PCa foci).TMA-TransBTS (2025) adopts multi-scale self-attention (TMSM) that uses shared queries and multi-scale keys/values to capture PCa scale differences but relies on depthwise convolution for scale mapping, leading to insufficient integration of local and global features.

#### 2.7.4 Task scope and clinical adaptability

Our model supporting multi-task learning (segmentation+grading) among the four compared methods. The segmentation task provides precise spatial constraints for grading (e.g., PCa volume, location), while the grading task provides clinical semantic guidance for segmentation (e.g., prioritizing regions with high malignant potential). This aligns with the clinical decision-making process (radiologists first locate PCa and then assess their malignancy). The model is specifically designed for bpMRI of PCa, fully exploiting the complementary information of T2WI (anatomical structure) and DWI (diffusion characteristics), and is suitable for clinical application in resource-limited institutions due to the cost and time advantages of bpMRI.3D TransUNet/TMA-TransBTS/DCTNet are single-task segmentation models, only outputting PCa masks without providing clinical significance assessment. They require additional post-processing or separate models for PCa grading, failing to meet the efficiency needs of clinical practice for PCa diagnosis. These models are designed for general organ/PCa segmentation or specific PCa segmentation of other parts (breast, brain), without targeted optimization for the imaging characteristics and clinical diagnosis requirements of PCa.

## 3 Results

### 3.1 Data and annotation

The information we utilized was gathered from PCa patients at Haikou People's Hospital (Affiliated Haikou Hospital of Xiangya Medical College, Central South University) between 2013 and 2016. The doctor performed MPMRI on all patients and identified suspected cancer. Using the GE3.0 T Signa HDX MRI scanner from the United States, a composite eight channel abdominal phased array coil is used as the receiving coil, without the use of a rectal coil. During this period, prostate biopsies were performed and diagnosed as PCa. Pathological diagnosis is conducted by pathologists certified by the hospital board of directors according to the Grissom grading system. The local cohort comprised 98 PCa patients with a median age of 68 years (range, 52–83 years). The median prostate-specific antigen (PSA) level was 18.5 ng/mL (range, 4.2–67.3 ng/mL), and the Gleason score distribution was as follows: 18 cases (18.4%) with a score of 6, 52 cases (53.1%) with a score of 7, and 28 cases (28.6%) with a score of 8–10. In terms of PCa spatial distribution, the majority of PCa were located in the peripheral zone (76 cases, 77.55%), with the remaining 22 cases (22.45%) situated in the transition zone; the median PCa size was 1.8 cm (range, 0.6–4.3 cm). Regarding the PI-RADS classification balance, the dataset included 41 cases (41.84%) with PI-RADS scores ≤ 3 and 57 cases (58.16%) with scores ≥4. To mitigate this minor class imbalance and ensure balanced gradient updates during model training, the Synthetic Minority Oversampling Technique (SMOTE) was applied to augment the PI-RADS ≤ 3 subset by generating synthetic samples based on feature similarity. The experimental data showed that the voxel size of T2WI images corresponding to the initial diagnosis of PCa in 98 patients was 512 × 512 × 22, and the actual scanning field FOV of the corresponding patients was 240 mm × 240 mm, with a thickness of 4 mm. The voxel size corresponding to DWI is 256 × 256 × 22. The corresponding patient's actual scanning field of view FOV is 400 mm × 512 × 400 mm, with a thickness of 4 mm. Please note that the dataset used has passed the ethical review of relevant hospitals and obtained the consent of informed patients.

This dataset is composed of two senior urologists with 10-20 years of experience in prostate MRI examinations, who manually annotate prostate and tumor areas. Before annotation, two experts personally met to participate in image annotation training. All image annotation work is independently carried out by two experts. The quantitative results of inter expert and intra expert consistency assessments for this dataset are summarized in Table 1, where *A*_*i*_(*i* = 1, 2) represents the i-th segmentation result of the first expert and *B*_*i*_(*i* = 1, 2) represents the i-th segmentation result of the second expert. *A*_1 & 2_ and *B*_1 & 2_ represent the intersection of the segmentation results of the first and second experts, respectively. Calculate the differences between experts by considering the intersection between each expert. Correlation coefficient (CC) is a statistical indicator used to measure the degree of linear correlation between two variables. Its value ranges from -1 to 1, obtained through a specific calculation formula, reflecting the degree and direction of correlation between variables. The closer the value of CC is to 1, the higher the correlation between the annotated images of different experts, that is, using this annotated image as a label for the training set will be more accurate. As shown in Table 1, the comparison results within and between experts show a very high CC, indicating a very high linear correlation between different experts and the same expert. The average Jaccard (>90%) and AAD (Equation 10) (AAD < 5%) indicate a high degree of consistency among and within experts. Finally, select the intersection segmentation labels of the two experts as the truth labels for the experiment. In addition, the two experts separately annotated all images with PI-RADS scores (PI-RADS ≤ 3 and PI-RADS ≥ 4), as shown in Table 1. Accuracy (ACC) is a commonly used evaluation metric in classification tasks, which measures the proportion of correctly classified samples by a classifier to the total number of samples. ACC is used to compare the accuracy of PI-RADS scores annotated by different experts with the actual situation of patients. The higher the ACC, the more accurate the results annotated by experts. Of course, ACC also serves as a measure of the accuracy of our model's automatic scoring of PI-RADS. Improving the performance of ACC can better predict the true PI-RADS score. By calculating the ACC, the intersection classification label of the two experts was obtained as the best annotation result.


$$\begin{array}{l} CC=\frac{\overset{\text{n}}{\underset{\text{i}=1}{\sum }}\left({\text{A}}_{\text{i}}-\bar{\text{A}}\right)\left({\text{B}}_{\text{i}}-\bar{\text{B}}\right)}{\sqrt{\overset{\text{n}}{\underset{\text{i}=1}{\sum }}{\left({\text{A}}_{\text{i}}-\bar{\text{A}}\right)}^{2}}\sqrt{\overset{\text{n}}{\underset{\text{i}=1}{\sum }}{\left({\text{B}}_{\text{i}}-\bar{\text{B}}\right)}^{2}}} \end{array}$$ (9)

$$\begin{array}{l} AAD=\frac{1}{\text{N}}\overset{\text{N}}{\underset{\text{i}=1}{\sum }}|\mathrm{Area}\left({\text{A}}_{\text{i}}\right)-\mathrm{Area}\left({\text{B}}_{\text{i}}\right)| \end{array}$$ (10)

$$\begin{array}{l} ACC=\frac{TP+TN}{FP+FN+TP+TN} \end{array}$$ (11)

where where A_i_ represents the segmentation result of the *i*−*th* expert (for inter/intra-expert consistency analysis), B_i_ represents the segmentation result of the *j*−*th* expert (for inter/intra-expert consistency analysis), $\bar{\text{A}}$ represents the mean value of expert A's segmentation results (for correlation coefficient calculation), $\bar{\text{B}}$ represents the mean value of expert B's segmentation results (for correlation coefficient calculation). *TP* represents the true positive: number of voxels/samples correctly predicted as target (prostate/PCa for segmentation; PI-RADS ≥ 4 for grading). *TN* represents the true negative: number of voxels/samples correctly predicted as non-target (background for segmentation; PI-RADS ≤ 3 for grading). *FP* represents the false positive: number of voxels/samples incorrectly predicted as target. *FN* represents the false negative: number of voxels/samples incorrectly predicted as non-target. To verify the generalizability of the proposed model, an independent external validation dataset PROMISE12 (26) was used, which comprises 50 cases of prostate MRI data. This dataset is independent of the original local dataset in terms of institution, scanning devices, and patient population, with multi-center and multi-vendor data and different acquisition protocols (e.g., differences in slice thickness, with/without endorectal coil). The PROMISE12 dataset only includes transversal T2-weighted MR images of the prostate, and the model's segmentation performance on this external dataset was evaluated using the same metrics (CC, HD, DSC, MIoU) as the original experiment to verify the universality of the model.

**Table 1:** Quantitative evaluation results within and between experts.

Comparison method	CC	AAD (mm)	Jaccard (%)	ACC (%)
*Exp*_A1_−*Exp*_A2_	0.997	3.15	94.23	99.45
*Exp*_B1_−*Exp*_B2_	0.998	3.85	95.44	99.89
*Exp*_A1&2_−*Exp*_B1&2_	0.996	4.26	92.37	97.58

### 3.2 Data pre-processing

Our experimental data showed that the voxel size of T2WI images corresponding to the initial diagnosis of PCa in 98 patients was 512 × 512 × 22, and the actual scanning field FOV of the corresponding patients was 240 mm × 240 mm, with a thickness of 4 mm. The voxel size corresponding to DWI is 256 × 256 × 22. The corresponding patient's actual scanning field of view FOV is 400 mm × 400 mm, with a thickness of 4 mm. Apply affine alignment to avoid any displacement in T2WI and DWI scans. Affine registration is a commonly used T2WI and DWI tumor volume rendering method by doctors. For simplicity, affine transformation was chosen in this study. In addition, sample augmentation was performed on all training data, flipping each prostate T2WI and DWI image from left to right and from top to bottom; And rotated the image by 90°, 180°, and 270°; Flipping preserves the visual structure. After data augmentation, the sample size can train deep learning models to avoid overfitting. In the experiment, we consider dividing the datasets of the two modalities into training and testing sets with a ratio of 4:1, respectively. And conduct 5-fold cross validation in the experiment, and then quantitatively evaluate the results of the test data on average.

For data normalization, T2WI images were processed with prostate-restricted z-score normalization: after manually extracting the prostate region via annotations, the mean and standard deviation of intensities within this region were calculated to normalize the entire volume, followed by clipping extreme values to the range [–3, 3] for noise suppression. For DWI images, they were first converted to apparent diffusion coefficient (ADC) maps using MRIcroGL (with b-values set to 0 and 1,000 s/mm^2^), then masked with prostate annotations to exclude non-prostate tissue; min-max scaling was applied based on the 5th and 95th percentiles of ADC values within the prostate region, mapping the values to the range [0, 1]. Prostate-centered 3D cropping was adopted as the cropping strategy: the bounding box of the prostate annotation mask was first extracted along the x, y, and z axes, then expanded by 10 voxels on all sides to retain contextual information critical for PCa boundary detection, with zero-padding applied if the expanded box exceeded the original volume dimensions; the average cropped size was 180 × 180 × 20 voxels, with a range of 160 × 160 × 18 to 200 × 200 × 22 voxels. Interpolation was implemented using SimpleITK: linear interpolation was used for T2WI images, while nearest-neighbor interpolation was applied for DWI images, and both modalities were resampled to a uniform voxel size of 1.0 × 1.0 × 4.0 mm^3^ to ensure consistent feature extraction across modalities.

To optimize training efficiency and avoid background dominance, a targeted patch sampling strategy is adopted: fixed 3D patches of 64 × 64 × 16 voxels are sampled from prostate-centered cropped volumes, with a sliding window stride of 32 voxels (50% overlap) for the training set and 16 voxels (75% overlap) for validation/test sets to reduce edge artifacts during inference. Patches with prostate+PCa pixels accounting for less than 5% are discarded, and during training, 70% of patches are sampled from PCa-containing regions to address PCa sparsity, with 30% from non-PCa prostate regions to maintain anatomical context balance.To mitigate severe class imbalance (background ≈ 90%, prostate ≈ 8%, PCa ≈ 2%), a mixed loss function for segmentation tasks is used (Dice loss with weight η_*D*_=0.9 and Focal loss with weight η_*F*_=0.1), and the Focal loss assigns a higher weight (φ=0.2) to tumor pixels to emphasize their learning. In addition, anatomy-preserving data augmentations (flipping, rotation, shearing, Gaussian noise, gamma correction) are applied exclusively to the training set to expand minority class samples, and the multitask loss function (L_MMTL) adaptively updates uncertain weights α_*seg*_ and α_*cls*_ through network learning to implicitly prioritize harder tasks like PCa segmentation.

### 3.3 Evaluation metrics

Five performance evaluation metrics were used to quantitatively evaluate the segmentation results of prostate and PCa, measuring model performance, including CC defined as Equation 9, HD defined as Equation 12, Equation 13 and Equation 14 are specific calculation methods for the parameters in Equation 12. DSC defined as Equation 15, and Mean Intersection over Union (MIoU) defined as Equation 16:


$$\begin{array}{l} HD=\frac{1}{\text{n}}\overset{\text{n}}{\underset{\text{i}=1}{\sum }}\max\left(h\left(\text{p}\left({\text{x}}_{\text{i}}\right),\text{g}\left({\text{x}}_{\text{i}}\right)\right),h\left(\text{g}\left({\text{x}}_{\text{i}}\right),\text{p}\left({\text{x}}_{\text{i}}\right)\right)\right) \end{array}$$ (12)

$$\begin{array}{l} h\left(\text{p}\left({\text{x}}_{\text{i}}\right),\text{g}\left({\text{x}}_{\text{i}}\right)\right)=\underset{{\text{a}}_{\text{i}}\in \text{p}\left({\text{x}}_{\text{i}}\right)}{\max}\left\{\underset{{\text{b}}_{\text{i}}\in \text{g}\left({\text{x}}_{\text{i}}\right)}{\min}||{\text{a}}_{\text{i}}-{\text{b}}_{\text{i}}||\right\} \end{array}$$ (13)

$$\begin{array}{l} h\left(\text{g}\left({\text{x}}_{\text{i}}\right),\text{p}\left({\text{x}}_{\text{i}}\right)\right)=\underset{{\text{b}}_{\text{i}}\in \text{g}\left({\text{x}}_{\text{i}}\right)}{\max}\left\{\underset{{\text{a}}_{\text{i}}\in \text{p}\left({\text{x}}_{\text{i}}\right)}{\min}||{\text{b}}_{\text{i}}-{\text{a}}_{\text{i}}||\right\} \end{array}$$ (14)

$$\begin{array}{l} DSC=\frac{\overset{n}{\underset{i=1}{\sum }}|p\left(x_{i}\right)\cap g\left(x_{i}\right)|}{\overset{n}{\underset{i=1}{\sum }}\frac{1}{2}\left(\left|p\left(x_{i}\right)|+|g\left(x_{i}\right)\right|\right)}=\frac{\overset{n}{\underset{i=1}{\sum }}2|p\left(x_{i}\right)\cap g\left(x_{i}\right)|}{\overset{n}{\underset{i=1}{\sum }}|p\left(x_{i}\right)|+|g\left(x_{i}\right)|} \end{array}$$ (15)

$$\begin{array}{l} \text{MIoU}=\frac{\text{TP}}{\text{FP}+\text{FN}+\text{TP}}\times 100\% \end{array}$$ (16)

where *p*(*x*_*i*_) represents predicted probability (or predicted region) of the *i*−*th* pixel/voxel. *g*(*x*_*i*_) represents the ground-truth label of the *i*−*th* pixel/voxel (segmentation: 1 = target, 0 = background). *TP*, *TN*, *FP* and *FN* respectively represent true positive, true negative, false positive, and false negative.

Five performance evaluation metrics were used to quantitatively evaluate the automatic grading results of prostate PI-RADS, including calculating ACC definition Equation 11, precision (PRE) definition Equation 17, recall (REC) as Equation 18, specificity (SPE) as Equation 19, and F1 score (F1 score, F1) as Equation 20 to evaluate performance.


$$\begin{array}{l} \text{PRE}=\frac{\text{TP}}{\text{FP}+\text{TP}}\times 100\% \end{array}$$ (17)

$$\begin{array}{l} \text{REC}=\frac{\text{TP}}{\text{FN}+\text{TP}}\times 100\% \end{array}$$ (18)

$$\begin{array}{l} \text{SPE}=\frac{\text{TN}}{\text{TN}+\text{FP}}\times 100\% \end{array}$$ (19)

$$\begin{array}{l} \text{F}1=2\times \frac{\text{PRE}\times \text{REC}}{\text{PRE}+\text{REC}}\times 100\% \end{array}$$ (20)

where *TP*, *TN*, *FP* and *FN* respectively represent true positive, true negative, false positive, and false negative.

### 3.4 Implementation details

This experiment was conducted on hardware settings including Intel Xeon CPU and 16 GB NVIDIA Tesla V100 PCIe GPU. The proposed model is implemented on Python 3.7 and PyTorch platforms and trained using a 5-fold cross validation strategy. The backbone network of the proposed network structure is V-Net (25). The Adam optimizer is used to randomly optimize the proposed model and other models, with an initial learning rate of 0.0001 and weight decay of 0.00002. In addition, set the block size to 8 and the number of training sessions to 300.

The model is trained for 300 epochs with a batch size of eight (optimized for 16 GB NVIDIA Tesla V100 GPU memory) using the Adam optimizer (initial learning rate = 10^−4^, β_1_ = 0.9, β_2_ = 0.999, weight decay = 2 × 10^−5^). The learning rate is adjusted via ReduceLROnPlateau with a patience of 10 epochs, a reduction factor of 0.5, and a minimum learning rate of 10^−6^. Convergence is determined by dual-task performance stability: early stopping is triggered if the combined validation score (weighted sum of prostate DSC + PCa DSC + PI-RADS ACC) does not improve for 20 consecutive epochs, with secondary criteria requiring validation DSC ≥91% for prostate, ≥87% for PCa, and ACC ≥93% for PI-RADS grading; the final model is selected as the epoch with the highest combined validation score (average convergence epoch: 268 ± 12 across 5 folds). Hardware specifications are standardized as follows: Intel Xeon Gold 6230 CPU (24 cores), 1 × 16 GB NVIDIA Tesla V100 GPU (CUDA Compute Capability 7.0), 256 GB DDR4-2933 RAM, and 4 TB NVMe SSD; the software environment uses Ubuntu 20.04 LTS, Python 3.7.13, PyTorch 1.12.1 (compiled with CUDA 11.6), cuDNN 8.4.1, and fixed versions of critical dependencies (Scikit-learn 1.0.2, SciPy 1.7.3, NumPy 1.21.6, etc).

### 3.5 Ablation study

#### 3.5.1 Model parameter ablation experiment

In the network structure designed, there are four very important hyperparameters φ, γ, η_*D*_ and η_*F*_ in the Loss function. To investigate the impact of different parameter settings on network performance, the control variable method was used to train the network with different parameter values. As shown in Table 2, first control (γ, η_*D*_, η_*F*_) to (0.5,0.5,0.5). Exploring the impact of increasing or decreasing weights in φ on network performance, φ is set to 0.1, 0.2, and 0.3. Based on the evaluation indicators DSC and HD, determine the optimal parameter ratio, and then control (γ, η_*D*_, η_*F*_) to (0.2, 0.5, 05) to explore the impact of increasing or decreasing γ weights on network performance. Therefore, set γ to 0.5, 1, and 2. Based on the evaluation indicators DSC and HD, determine the optimal parameter ratio, then control (φ, γ) to be the optimal parameter ratio and keep it constant. Adjust the parameter ratios of (η_*D*_, η_*F*_) to (0.1, 0.9) and (0.9, 0.1), to increase or reduce the weight of DCE loss or Focal loss, and thus evaluate network performance. Table 2 shows that the proposed method achieves the lowest HD and highest DSC for prostate and PCa segmentation when φ is set to 0.2, γ is set to 1, and (η_*D*_, η_*F*_) are set to (0.9,0.1), respectively. To enhance the rigor and credibility of experimental conclusions, we have conducted paired *t*-tests for continuous segmentation metrics (DSC, HD, CC, MIoU) and Wilcoxon rank-sum tests for categorical grading metrics (ACC, PRE, REC, SPE, F1) for all comparative experiments. We also calculated the 95% confidence intervals (95% CI) for all key metrics to reflect the stability of the results. A significance level of α=0.05 is used for all tests. *p* < 0.05 indicates significant difference, *p* < 0.001 indicates extremely significant difference. All results are reported as mean ± standard deviation (SD) to reflect the stability of the experimental results.

**Table 2:** The impact of different parameter settings on network performance.

Parameter				Prostate segmentation			PCa segmentation		
φ	γ	η_*D*_	η_*F*_	HD (mm) (mean ± SD)	DSC (%) (mean ± SD)	*P*-value	HD (mm) (mean ± SD)	DSC (%) (mean ± SD)	*P*-value
0.1	0.5	0.5	0.5	0.6025 ± 0.42	87.38 ± 1.25	*p* < 0.001	0.8312 ± 0.51	83.55 ± 1.32	*p* < 0.001
0.2	0.5	0.5	0.5	0.5715 ± 0.38	88.66 ± 1.18	*p* < 0.001	0.7827 ± 0.47	84.43 ± 1.27	*p* < 0.001
0.3	0.5	0.5	0.5	0.6218 ± 0.45	86.53 ± 1.31	*p* < 0.001	0.9521 ± 0.58	82.16 ± 1.38	*p* < 0.001
0.2	1	0.5	0.5	0.3218 ± 0.29	90.85 ± 1.05	*p* = 0.002	0.6247 ± 0.41	87.61 ± 1.15	*p* = 0.003
0.2	2	0.5	0.5	0.3671 ± 0.32	90.14 ± 1.09	*p* = 0.005	0.6915 ± 0.44	86.82 ± 1.21	*p* = 0.007
0.2	1	0.1	0.9	0.4627 ± 0.37	89.65 ± 1.12	*p* < 0.001	0.7218± 0.46	85.27 ± 1.24	*p* < 0.001
**0.2**	**1**	**0.9**	**0.1**	**0.2854** **±** **0.25**	**92.28** **±** **0.98**	–	**0.5746** **±** **0.38**	**88.73** **±** **1.08**	–

The bold values represent the best experimental results.

#### 3.5.2 Network structure ablation study

**Figure 6:**
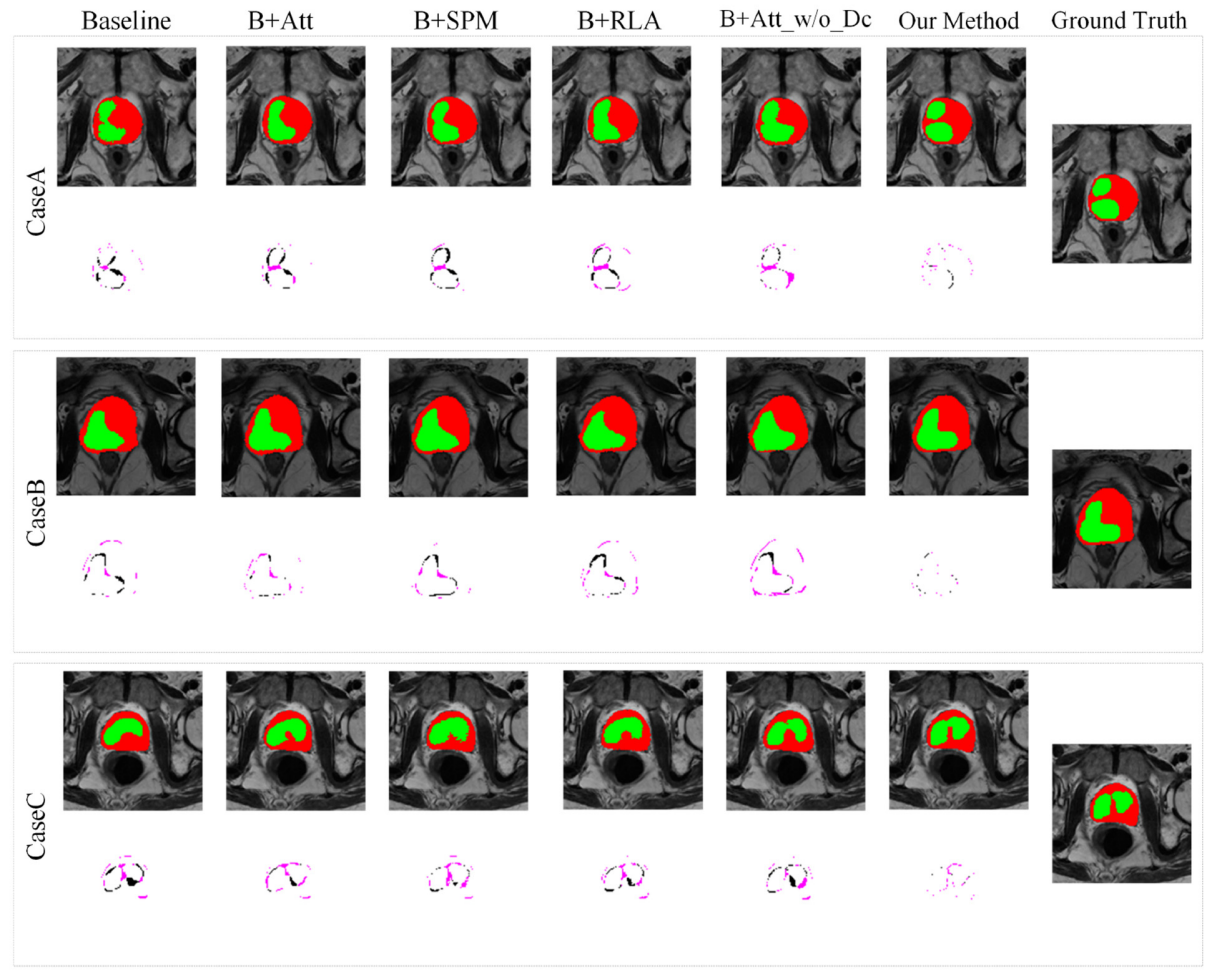
Visual ablation analysis of prostate and PCa segmentation.

To demonstrate the accuracy of the designed network structure for prostate and PCa region segmentation, attention and SPM were added to the input baseline (V-Net), and different modules were aggregated on the baseline. The detailed results are shown in Tables 3–5. Adding the proposed attention (B+Att) and SPM (B+SPM) to the baseline significantly improved the five evaluation indicators. Especially for the dual visual attention module, compared to the baseline, the DSC of prostate segmentation increased by 5.62% (89.38% vs. 83.76%), and the DSC of PCa segmentation increased by 9.91% (86.25% vs. 76.34%). Therefore, the combination of standard convolution and dilated convolution in the attention module plays a crucial role in prostate and PCa segmentation by providing detailed information. In addition, attention without dilated convolution (B+Att_w/o_Dc) and only residual linear attention (RLA) layer (B+RLA) were added to the baseline for comparison with the designed dual visual attention and RLA segmentation performance. As shown in Tables 3, 4, the designed module achieved better segmentation performance in the prostate and PCa regions. The method proposed involves three modules (attention, SPM, and RLA) working together to complete the segmentation task. As shown in Tables 3, 4, the method proposed has further improved segmentation performance compared to adding only one module. Compared to the baseline, the method proposed improved the DSC value of prostate MRI segmentation by 8.52% (92.28% vs. 83.76%), and the DSC value of PCa bpMRI segmentation by 12.39% (88.73% vs. 76.34%). In the classification task of PI-RADS automatic scoring, the proposed method has further improved the performance of segmentation classes compared to adding only one module. Compared to the baseline, as shown in Table 5, the proposed method improved ACC, PRE, REC, SPE, and F1 in PI-RADS automatic classification by 8.82%, 9.38%, 8.01%, 8.12%, and 8.72%, respectively. Therefore, the dual visual attention, SPM, and RLA proposed all play important roles in target region segmentation and are indispensable. The improvement rate of PCa segmentation performance is greater than that of prostate segmentation, indicating that the proposed method is beneficial for segmenting regions with irregular shapes, blurred boundaries, unfixed positions, and smaller targets. The results of the method proposed and the CC of the truth labels are both greater than 0.9 (0.9734 and 0.9458), indicating a high correlation and consistency between the automatic segmentation results of the prostate and PCa and the manual segmentation results of experts. The decrease in average HD value also supports the superior segmentation performance of the method proposed. For the PI-RADS automatic grading (PI-RADS ≤ 3 and PI-RADS ≥ 4) task, the addition of each module improves the classification performance, indicating the effectiveness of the proposed method. Figure 6 further illustrates the qualitative comparison results of the model for segmenting bpMRI images of the prostate and PCa regions by adding different modules and true value labels on the baseline. Each case contains a segmentation result of a 2D slice image and a difference map from the true value label. The red and green colors in the 2D slice image represent the prostate and PCa regions, respectively. The purple color in the difference chart represents the prostate segmentation error, while the black color represents the PCa segmentation error. Each column is the segmentation result of different methods. From left to right: Baseline, add attention (B+Att), add SPM (B+SPM), add RLA layer (B+RLA), add dual visual attention without dilated convolution ((B+Att_w/o_Dc) and our method, with truth labels on the far right. Adding other components to the baseline can improve the segmentation performance of prostate and PCa to some extent, but relying solely on one module cannot achieve accurate segmentation. Only when multiple modules cooperate with each other can more accurate boundary segmentation be achieved. Compared to the baseline and single module, the model in this paper achieves significant improvements in all indicators, and the comparison with the baseline has a *p*-value < 0.001 (extremely significant difference). Moreover, the improvement in PCa segmentation is significantly higher than that in the prostate, validating the adaptability of the model to difficult-to-segment areas.

**Table 3:** Ablation study of different components of prostate segmentation modules in networks.

Segmentation method	Prostate segmentation				
	CC (mean ± SD)	HD (mm) (mean ± SD)	DSC (%) (mean ± SD)	MIoU (%) (mean ± SD)	*P*-value
Baseline	0.9285 ± 0.12	0.8761 ± 0.68	83.76 ± 1.52	73.65 ± 1.85	*p* < 0.001
B+Att	0.9673 ± 0.09	0.4438± 0.42	89.38 ± 1.31	80.19 ± 1.62	*p* = 0.003
B+SPM	0.9585 ± 0.10	0.5165 ± 0.47	87.92 ± 1.38	78.78 ± 1.68	*p* = 0.001
B+RLA	0.9572 ± 0.11	0.5826 ± 0.51	87.14 ± 1.42	77.84 ± 1.73	*p* < 0.001
B+Att_w/o_Dc	0.9519 ± 0.12	0.6163± 0.53	86.64 ± 1.45	76.63 ± 1.78	*p* < 0.001
**Our method**	**0.9734** **±** **0.08**	**0.2854** **±** **0.25**	**92.28** **±** **0.98**	**85.16** **±** **1.45**	–

The bold values represent the best experimental results.

**Table 4:** Ablation study of different components of PCa segmentation modules in networks.

Segmentation method	PCa segmentation				
	CC (mean± SD)	HD (mm) (mean ± SD)	DSC (%) (mean ± SD)	MIoU (%) (mean ± SD)	*P*-value
Baseline	0.8652 ± 0.18	1.2736 ± 0.89	76.34± 1.65	61.67 ± 1.98	*p* < 0.001
B+Att	0.9377 ± 0.11	0.6353 ± 0.45	86.25 ± 1.32	75.61 ± 1.65	*p* = 0.005
B+SPM	0.9283 ± 0.12	0.6732 ± 0.48	84.56 ± 1.38	73.94 ± 1.71	*p* = 0.002
B+RLA	0.9136 ± 0.14	0.9544± 0.65	81.35 ± 1.45	68.28 ± 1.85	*p* < 0.001
B+Att_w/o_Dc	0.9018 ± 0.15	1.0685 ± 0.72	80.89± 1.48	67.44 ± 1.89	*p* < 0.001
**Our method**	**0.9458** **±** **0.09**	**0.5746** **±** **0.38**	**88.73** **±** **1.08**	**80.77** **±** **1.52**	–

The bold values represent the best experimental results.

**Table 5:** Ablation analysis of different components of modules in networks in PI-RADS grading.

Classification method	PI-RADS grading					
	ACC (%) (mean ± SD)	PRE (%) (mean ± SD)	REC (%) (mean ± SD)	SPE (%) (mean ± SD)	F1 (%) (mean ± SD)	*P*-value
Baseline	85.74 ± 2.92	86.36 ± 2.85	89.83 ± 2.72	90.15 ± 3.68	88.06 ± 1.78	*p* < 0.001
B+SPM	87.43 ± 2.83	89.02 ± 3.76	92.55± 2.59	93.89± 2.52	90.75 ± 1.65	*p* = 0.003
B+RLA	88.85 ± 1.75	90.64 ± 1.68	93.78 ± 1.51	95.39 ± 2.43	92.18 ± 1.57	*p* = 0.001
B+RLA+HLFF	93.16 ± 1.42	92.82 ± 1.53	95.66 ± 1.35	96.57 ± 1.31	94.22 ± 1.45	*p* = 0.021
**Our method**	**94.56** **±** **1.28**	**95.74** **±** **1.36**	**97.84** **±** **1.15**	**98.27±** **1.08**	**96.78** **±** **1.25**	–

The bold values represent the best experimental results.

#### 3.5.3 Different modal ablation study

The proposed method is a dual-mode fusion network, where the encoder stage inputs images of different modalities. Input T2WI and DWI prostate and PCa MRI images into the constructed network encoder. PCa is affected by the degree and region of cancerous transformation, and exhibits various characteristics, sizes are not fixed, and boundaries are blurred on T2WI and DWI. The degree of realization varies significantly in different modalities. From Figure 1, it can be seen that some PCa only shows significant performance on T2WI, while others only show significant performance on DWI. Therefore, it is necessary to consider the image features of both modalities simultaneously in order to better train the network. DWI scanning has good spatial intensity resolution for PCa, especially PCa tissues. T2WI images can better reflect the anatomical structure of organs and tissues, accurately locate PCa, and display morphology. In order to confirm that the dual-mode fusion method proposed is superior to the single-mode network, a comparison was made between the two modal fusion methods with only T2WI and DWI inputs, while keeping other modules unchanged. Most current methods extract feature maps through two non interfering branches, which cannot benefit each other during the feature extraction process. This chapter uses two encoder blocks separated from each layer to extract information, and the outputs of the two encoder blocks share a subsampling layer to obtain the same spatial features. The convolutional kernel promotes information exchange between T2WI and DWI channels, enhancing the learning ability of the network.

**Figure 7:**
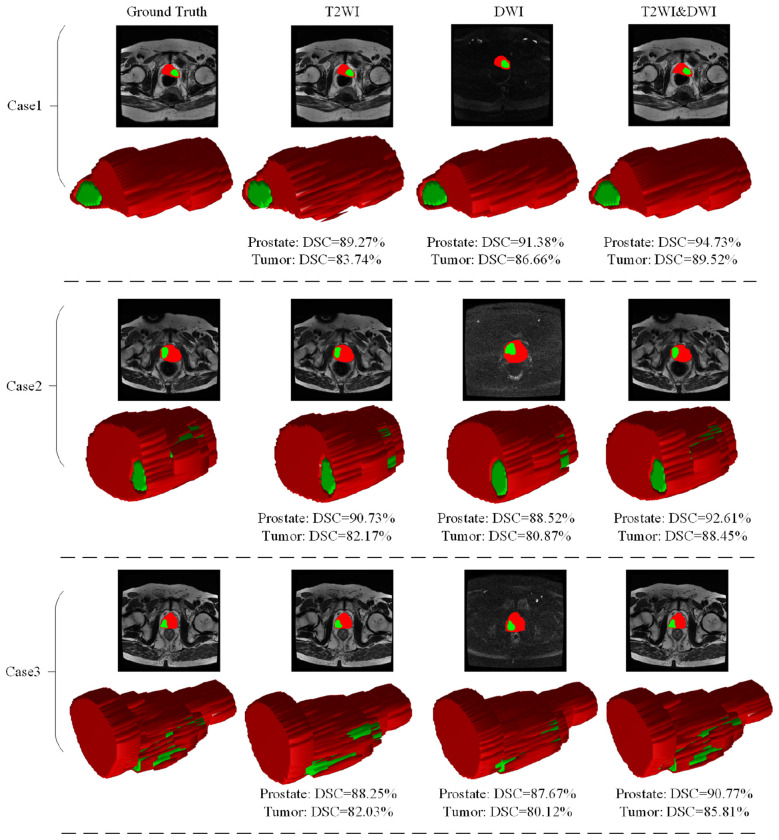
3D visualization of prostate and PCa segmentation results with different modalities.

Figure 7 shows the 3D visualization of prostate and PCa segmentation results with different modalities. On 2D imaging, the red area represents the prostate region and the green area represents the PCa region. The red area on 3D imaging is the prostate area, and the green area is the PCa area. We also provided DSC values for prostate and PCa segmentation for three independent patients. From Figure 7, whether it is 2D imaging or 3D imaging, the method proposed has higher consistency with the truth label in terms of the size, position, and angle of the segmented target. For Case 2 with DSC close to the mean, the DSC values of multimodal MRI segmentation of the prostate increased by 1.88% (92.61% vs. 90.73%) and 4.09% (92.61% vs. 88.52%) compared to the other two unimodal methods. The DSC values of PCa segmentation increased by 6.28% (88.45% vs. 82.17%) and 7.58% (88.45% vs. 80.87%), respectively. For Case 3 with difficulty in automatic segmentation, the DSC values of multimodal MRI segmentation of the prostate increased by 2.52% (90.77% vs. 88.25%) and 3.1% (90.77% vs. 87.67%) compared to the other two single-mode methods. The DSC values of PCa segmentation increased by 3.78% (85.81% vs. 82.03%) and 5.69% (85.81% vs. 80.12%), respectively. From this, it can be seen that the method proposed has a greater improvement in segmentation performance for smaller and more difficult to segment target regions. For Case 1, where automatic segmentation is relatively easy, the DSC values of multimodal MRI segmentation of the prostate increased by 5.46% (94.73% vs. 89.27%) and 3.35% (94.73% vs. 91.38%) compared to the other two single-mode methods. The DSC values of PCa segmentation increased by 5.78% (89.52% vs. 83.74%) and 2.86% (89.52% vs. 86.66%), respectively. Therefore, for the automatic segmentation of prostate and PCa in MRI images, the dual-mode fusion input encoder proposed is superior to single-mode input, resulting in better segmentation results.

### 3.6 Comparative experiment

To verify the effectiveness of the proposed method, the segmentation comparison methods are divided into three categories:

**(1) Classic image segmentation networks:** 3D U-Net (27) and V-Net (25). 3D U-Net (27) has a symmetrical encoder decoder structure, where the feature map output by the decoder integrates more low-level features. V-Net (25) is a 3D U-Net deformable network of encoder decoder for prostate segmentation.

**(2) Automatic segmentation networks for prostate cancer:** CDA-Net (16) and BiAA-Net (28). CDA-Net (16) is a parallel expansion of U-Net to extract deep features for more accurate PCa automatic segmentation. BiAA-Net (28) uses a dual attention adversarial network consisting of a generator network and a discriminator network for automatic segmentation of PCa in MRI images.

**(3) Multimodal fusion segmentation networks:** MMC-L (29) and Coco Attention (30). MMC-L (29) shares downsampling blocks between two encoding branches to eliminate misleading features and achieve bimodal image segmentation. Coco Attention (30) can effectively utilize complementary information in weakly paired images for accurate tumor segmentation.

**(4) State-of-the-Art multimodal transformer architectures: 3D TransUNet** (20) is a general-purpose segmentation framework based on nnU-Net, designed for multi-organ/tumor segmentation (e.g., pancreatic tumors, hepatic vessels) without clinical grading functionality. DCTNet (21) use a two-branch separation-fusion strategy: dual CNN encoders extract modality-specific features, and a Transformer encoder extracts cross-modal shared features, followed by late fusion via the Cross-Modal Feature Fusion Module (CFM). TMA-TransBTS (22) adopt mid-stage cross-attention fusion via the 3D Multi-scale Cross-attention Module (TMCM), which replaces traditional skip connections in the decoder to fuse encoder multi-scale features with decoder features.

The comparison methods for classification are divided into two categories. CNN based models: GoogLeNet (31), VGG-16 (32), AlexNet (33), ResNet-50 (34), and DenseNet-121 (35). A CNN was constructed based on GoogLeNet for medical image classification research (31); Using pre trained VGG-16 for gastric image classification through fine-tuning (32); A deep learning framework was constructed based on AlexNet for cancer classification (33); Developed a novel artificial intelligence model combined with ResNet to diagnose colon polyps (34); A new multimodal 3D DenseNet model for IDH genotype classification using mpMRI data (35). Transformer based models: ViT (36), PiT (37), and CrossFormer (38). Propose a Transform for image patch sequences for image classification tasks (36); Starting from the successful design principles of CNN, the role and effectiveness of spatial dimension transformation in Transformer based architecture were studied (37); We have built a visual architecture called CrossFormer, based on ViT's dynamic position offset, to make the popular relative position offset suitable for variable size images (38).

#### 3.6.1 Quantitative analysis

Tables 6–9 show the segmentation performance scores of the proposed method and the comparison method in bpMRI images of the prostate and PCa regions. For the sake of fairness, all methods use the same training and testing data, and calculate segmentation performance scores using the same evaluation metrics. The proposed network underwent parameter ablation experiments, and similarly, other comparative methods also conducted parameter ablation experiments to obtain the best segmentation results of this method. The experimental results show that the proposed method achieves an average CC of 0.9734, HD of 0.2854 mm, DSC of 92.28%, and MIoU of 85.16% for prostate MRI segmentation. Among the comparison methods of classic 3D image segmentation networks, automatic prostate cancer segmentation networks, and multimodal fusion segmentation networks, the proposed method obtains the best metric values. Compared with the most classic image segmentation network 3D U-Net, the proposed method improves the CC, HD, DSC, and MIoU of prostate segmentation by approximately 0.045%, 0.59 mm, 8.52%, and 11.51%, respectively. In PCa segmentation, CC, HD, DSC, and MIoU increased by approximately 0.08%, 0.70 mm, 12.39%, and 16.1%, respectively. Compared with the latest prostate cancer segmentation method CDA Net, the proposed method improves the CC, HD, DSC, and MIoU of prostate segmentation by approximately 0.04, 0.29 mm, 6.72%, and 9.63%, respectively. In PCa segmentation, CC, HD, DSC, and MIoU were improved by approximately 0.04%, 0.18 mm, 6.1%, and 7.91%, respectively. All experiments use the same training/testing data and evaluation metrics (DSC, HD, MIoU for segmentation) to ensure fairness. All analyses are based on 5-fold cross-validation results of the proposed method and three comparative models (3D TransUNet, DCTNet, TMA-TransBTS), consistent with the experimental setting of the original manuscript. A significance level of α=0.05 is used for all tests. *p* < 0.05 indicates significant difference, *p* < 0.001 indicates extremely significant difference. All results report mean ± standard deviation (SD), consistent with the original manuscript's statistical presentation for other comparative methods. Our model outperforms 3D TransUNet, DCTNet, and TMA-TransBTS in all segmentation metrics with statistically significant differences (*p* < 0.05). Most comparisons show extremely significant differences (*p* < 0.005), confirming that the performance advantages are not due to random cross-validation fluctuations, but to the effectiveness of the proposed dual-modal fusion, dual visual attention, and multi-task learning design.

**Table 6:** The performance of prostate segmentation comparison between the proposed method and the comparative methods.

Segmentation method	Prostate segmentation				
	CC (mean ± SD)	HD (mm) (mean± SD)	DSC (%) (mean ± SD)	MIoU (%) (mean ± SD)	*P*-value
3D U-Net	0.9285 ± 0.12	0.8761 ± 0.68	83.76± 1.52	73.65 ± 1.85	*p* < 0.001
V-Net	0.9346 ± 0.11	0.4058 ± 0.39	86.17 ± 1.42	76.31± 1.72	*p* < 0.001
CDA-Net	0.9354 ± 0.14	0.5742 ± 0.46	85.56 ± 1.45	75.53± 1.76	*p* < 0.001
BiAA-Net	0.9248 ± 0.13	0.4664 ± 0.43	87.71 ± 1.36	79.48 ± 1.65	*p* = 0.002
MMC-L	0.9441 ± 0.09	0.4676 ± 0.43	89.35 ± 1.28	81.94 ± 1.58	*p* = 0.012
Coco attention	0.9572± 0.08	0.4063 ± 0.39	90.16 ± 1.21	83.16 ± 1.52	*p* = 0.023
**Our method**	**0.9734** **±** **0.06**	**0.2854** **±** **0.25**	**92.28** **±** **0.98**	**85.16** **±** **1.45**	–

The bold values represent the best experimental results.

**Table 7:** The performance of PCa segmentation comparison between the proposed method and the comparative methods.

Segmentation method	PCa segmentation				
	CC (mean ± SD)	HD (mm) (mean ± SD)	DSC (%) (mean ± SD)	MIoU (%) (mean ± SD)	*P*-value
3D U-Net	0.8652 ± 0.18	1.2736 ± 0.89	76.34 ± 1.65	64.67 ± 1.98	*p* < 0.001
V-Net	0.9166 ± 0.15	0.6844 ± 0.52	84.68 ± 1.48	74.14 ± 1.75	*p* = 0.018
CDA-Net	0.9033± 0.16	0.7525 ± 0.56	82.63 ± 1.55	72.86 ± 1.82	*p* = 0.009
BiAA-Net	0.9315 ± 0.11	0.5734 ± 0.38	85.12 ± 1.42	75.81 ± 1.68	*p* = 0.021
MMC-L	0.9356 ± 0.13	0.6128 ± 0.41	86.07 ± 1.36	76.19 ± 1.62	*p* = 0.034
Coco attention	0.9215 ± 0.12	0.6367 ± 0.44	86.75± 1.31	77.31 ± 1.58	*p* = 0.041
**Our method**	**0.9458** **±** **0.09**	**0.5746** **±** **0.38**	**88.73** **±** **1.08**	**80.77** **±** **1.52**	–

The bold values represent the best experimental results.

**Table 8:** Prostate segmentation: segmentation results of the SOTA methods and paired *t*-test results.

Segmentation method	CC (mean ± SD)	HD(mm) (mean ± SD)	DSC (%) (mean ± SD)	MIoU (%) (mean ± SD)	*p*-value
3D TransUNet	0.9512 ± 0.10	0.4125 ± 0.32	88.95 ± 1.12	80.28± 1.56	0.004
DCTNet	0.9467 ± 0.11	0.4583 ± 0.36	87.86 ± 1.25	79.15 ± 1.68	0.002
TMA-TransBTS	0.9586 ± 0.09	0.3569 ± 0.28	90.52 ± 0.98	82.64± 1.35	0.018
**Our method**	**0.9734** **±** **0.08**	**0.2854** **±** **0.25**	**92.28** **±** **0.98**	**85.16** **±** **1.45**	**–**

The bold values represent the best experimental results.

**Table 9:** PCa segmentation: segmentation results of the SOTA methods and paired *t*-test results.

Segmentation method	CC (mean ± SD)	HD(mm) (mean± SD)	DSC (%) (mean ± SD)	MIoU (%) (mean ± SD)	*p*-value
3D TransUNet	0.9289± 0.10	0.6218 ± 0.40	85.96 ± 1.15	76.89 ± 1.62	0.015
DCTNet	0.9195 ± 0.12	0.6874 ± 0.45	84.32 ± 1.32	74.56± 1.85	0.005
TMA-TransBTS	0.9367 ± 0.09	0.5983 ± 0.35	87.25 ± 1.02	78.14 ± 1.48	0.032
**Our method**	**0.9458** **±** **0.09**	**0.5746** **±** **0.38**	**88.73** **±** **1.08**	**80.77** **±** **1.52**	**–**

The bold values represent the best experimental results.

Table 10 shows the classification performance of PI-RADS automatic scoring, and compared to CNN based classification networks, the method proposed has achieved significant performance improvement. For example, the method proposed improves GoogLeNet by 11.11%, 10.88%, 10.47%, 8.52%, and 10.69% in ACC, PRE, REC, SPE, and F1, respectively. Compared to Transformer based classification networks, the method proposed has significantly improved classification results. For example, compared to the latest method CrossFormer, the method proposed has improved ACC, PRE, REC, SPE, and F1 by 1.79%, 1.63%, 2.11%, 1.2%, and 2.04%, respectively. It can be seen that the method proposed outperforms other methods in segmentation performance. The improvement in performance is mainly attributed to the proposed attention segmentation structure and Transformer providing more comprehensive visual information and high-level multi-scale feature information, thereby enhancing the performance of network learning. The analysis of ablation study is reflected in Tables 3–5. This further proves that the proposed method outperforms baseline networks, as well as other CNN based segmentation and classification networks and Transformer based classification networks in multiple tasks such as image classification and robustness evaluation. The clinical task collaborative design (segmentation + classification), early fine-grained SPM fusion, and dual visual attention mechanism proposed in this model are superior to the single segmentation task design of the Transformer method, shallow modal concatenation/mid-term cross-attention fusion, and are more suitable for the imaging characteristics of prostate cancer bpMRI.

**Table 10:** The performance of PI-RADS grading comparison between the proposed method and the comparative methods.

Classification method	PI-RADS grading					
	ACC (%) (mean± SD)	PRE (%) (mean ± SD)	REC (%) (mean ± SD)	SPE (%) (mean ± SD)	F1(%) (mean ± SD)	*P*-value
GoogLeNet	83.45± 1.98	84.86 ± 1.89	87.37 ± 1.78	89.15 ± 1.72	86.09 ± 1.82	*p* < 0.001
VGG-16	84.66± 1.92	85.34 ± 1.85	87.88 ± 1.75	90.67 ± 1.68	86.59± 1.78	*p* < 0.001
AlexNet	85.13 ± 1.88	86.25 ± 1.81	88.75 ± 1.71	90.72± 1.65	87.48 ± 1.75	*p* < 0.001
ResNet-50	85.46 ± 1.85	87.41 ± 1.76	89.38 ± 1.68	91.24 ± 1.61	88.38 ± 1.72	*p* < 0.001
DenseNet-121	87.18 ± 1.72	89.36 ± 1.68	90.76 ± 1.62	92.84 ± 1.53	90.05 ± 1.65	*p* < 0.001
ViT	90.46 ± 1.55	91.85 ± 1.51	93.27 ± 1.48	94.19 ± 1.42	92.55 ± 1.52	*p* = 0.002
PiT	92.53 ± 1.38	93.67 ± 1.42	94.16 ± 1.39	96.24 ± 1.28	93.91 ± 1.45	*p* = 0.011
CrossFormer	92.77 ± 1.35	94.11 ± 1.38	95.73 ± 1.31	97.07 ± 1.21	94.74 ± 1.39	*p* = 0.023
**Our method**	**94.56** **±** **1.28**	**95.74** **±** **1.36**	**97.84** **±** **1.15**	**98.27** **±** **1.08**	**96.78** **±** **1.25**	–

The bold values represent the best experimental results.

#### 3.6.2 External validation

To verify the universality of our proposed model, we utilized an independent external validation dataset, PROMISE12 (26), which comprises 50 cases of prostate MRI data. This dataset is independent of the original local dataset (Haikou People's Hospital, 2013–2016) in terms of institution, scanning equipment, and patient population, thereby ensuring the objectivity of external validation. PROMISE12 include a transversal T2-weighted MR image of the prostate. The training set is a representative set of the types of MR images acquired in a clinical setting. The data is multi-center and multi-vendor and has different acquistion protocols (e.g., differences in slice thickness, with/without endorectal coil). We evaluated the model's performance on the external dataset using the same metrics as the original experiment (CC, HD, DSC, MIoU for segmentation). The results are shown in Table 11, CC = 0.96, HD = 1.32 mm, DSC = 90.15%, MIoU = 83.27% (only 2.13% lower in DSC than the local dataset, indicating stable spatial localization ability). These results confirm that the model maintains high performance on an independent external dataset, effectively addressing the generalizability limitation of single-institution data.

**Table 11:** External prostate segmentation: segmentation results of the SOTA methods and paired *t*-test results.

Segmentation method	CC (mean± SD)	HD (mm) (mean ± SD)	DSC (%) (mean ± SD)	MIoU (%) (mean ± SD)	*p*-value
3D TransUNet	0.94 ± 0.25	3.67 ± 1.66	87.05 ± 1.04	80.48 ± 0.73	0.025
DCTNet	0.95 ± 0.87	2.67 ± 0.27	88.45± 0.79	81.16 ± 0.58	0.038
TMA-TransBTS	0.95 ± 0.38	1.35 ± 0.18	89.37 ± 0.45	82.36± 0.16	0.046
**Our method**	**0.96** **±** **0.54**	**1.32** **±** **0.12**	**90.15** **±** **0.02**	**83.27** **±** **0.07**	**–**

The bold values represent the best experimental results.

#### 3.6.3 Qualitative evaluation

**Figure 8:**
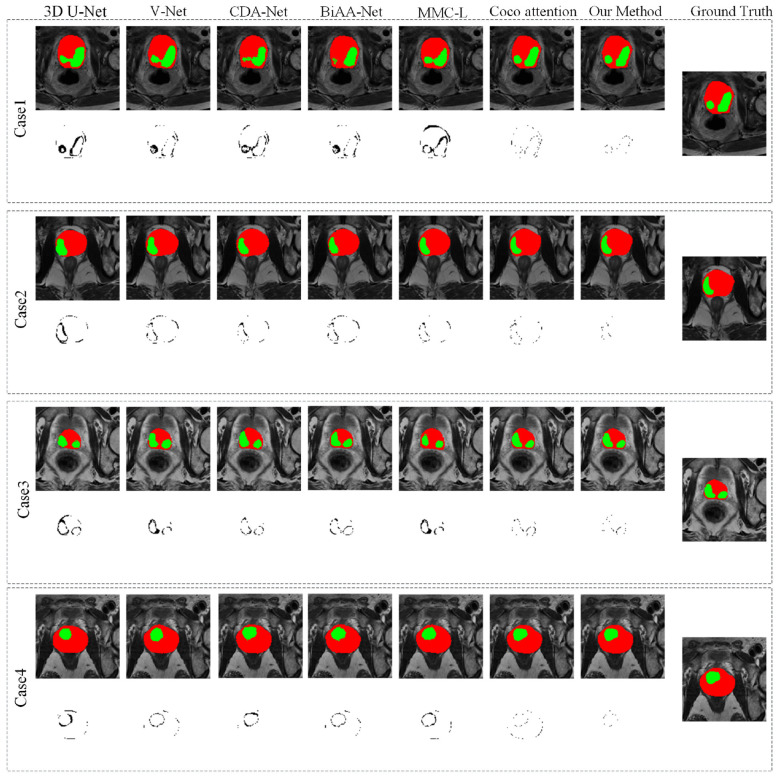
Comparison of different segmentation methods for MRI images of prostate and PCa regions.

Figure 8 further illustrates the qualitative comparison results of different methods and truth labels for bpMRI image segmentation in the prostate and PCa regions. Each case contains a segmentation result of a row of 2D slice images and a difference graph between the row and the truth label. The red and green in the 2D slice images represent the prostate and PCa regions, respectively. In the difference plot, black represents the segmentation error. Each column represents the segmentation results of different methods. From left to right: 3D U-Net, V-Net, CDA Net, BiAA-Net, MMC-L, Coco attention and our method, with truth labels on the far right. From the difference plot, it can be seen that compared with the other six methods, the method proposed can obtain segmentation results that are more consistent with the truth labels. These visualized prostate and PCa segmentation results further validate the effectiveness of the proposed method. As shown in Figure 8, for different methods, the prostate segmentation results of the four cases can all recognize this region. However, the other six methods still differ from truth labels in boundary segmentation. For example, in Case 1, there are two different cancerous regions, but the 3D-Unet, V-Net, CDA-Net, and MMC-L methods connect and divide the cancerous regions into one area, resulting in significant misclassification. Although the methods of BiAA-Net and Coco Attention successfully segmented the cancerous area into two regions, there were significant misclassifications at the boundaries. From the black area of the difference map (representing segmentation error), it can be observed that the segmentation error of our proposed method is the smallest.

Our method accurately identifies the prostate region while maintaining a high degree of overlap between the boundary and the truth labels, thanks to the effective capture of feature details and contextual information by the SPM, attention, and Transformer modules designed for multimodal image fusion. Input the feature volumes extracted by the encoder at different depths within the network into the attention module. In this way, each feature SPM block can absorb multimodal information at different resolutions below its depth. After mapping features of different sizes into the feature decoder, the proposed model is further enhanced. For PCa segmentation of small target areas, 3D U-Net, V-Net, channel attention, and dual attention methods have errors in identifying cancerous areas, which seriously affects the doctor's judgment of PCa. The method proposed can effectively identify PCa regions, whether it is a single cancerous region or multiple cancerous regions. Although BiAA Net and ConvLSTMs can also accurately identify PCa regions, there are still significant differences between important information such as the size and boundaries of the cancerous regions and the truth labels. In contrast, the method proposed can effectively identify the prostate region while accurately segmenting the boundaries of the cancerous area, which is highly consistent with the truth labels. This can confirm that the designed multimodal multitasking network structure exhibits strong performance in effectively extracting visual and multi-scale features, extracting detailed information, and deeper learning representations in prostate and PCa bpMRI image segmentation. The segmentation error of our proposed method is mainly reflected in the prostate cancer region, as shown in Figure 8. The black area of the error map for each case is mainly concentrated at the boundary of the cancerous tissue, which is a difficult part to segment accurately. We will continue to optimize the model to adapt to the boundary segmentation of different cancerous tissue regions and improve accuracy.

**Figure 9:**
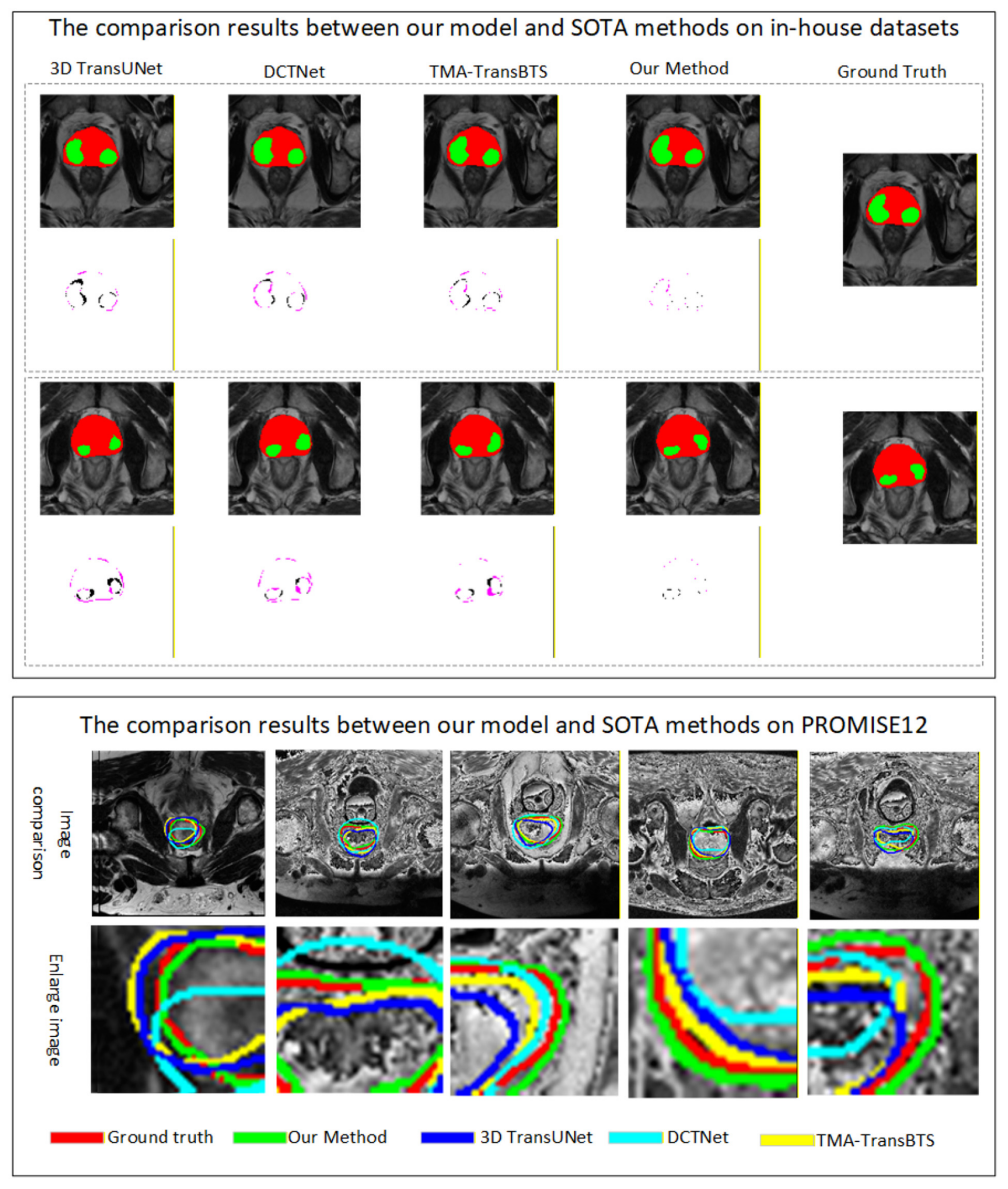
Comparison results between the proposed model and SOTA methods on internal and external datasets.

Figure 9 presents the performance comparison between our model and state-of-the-art methods on both internal and external datasets. The external dataset only involves the prostate region segmentation. To present more comprehensive experimental results, we visualize the segmentation outputs of different methods using line annotations. As shown in Figure 9, our model achieves superior segmentation performance on both datasets, maintaining a high degree of consistency with the ground truth labels.

### 3.7 Clinical implications

The proposed model's superior performance in segmentation and PI-RADS grading translates to tangible clinical benefits, addressing key pain points in PCa diagnosis and management:

(1) Reducing Inter-Observer Variability in Manual Segmentation: Manual delineation of prostate and PCa regions on 3D bpMRI slices is time-consuming and subjective, with inter-observer variability even among senior radiologists (AAD = 4.26 mm between experts). Our model's segmentation results show high consistency with the intersection gold standard of two expert annotations (CC = 0.9734 for prostate, 0.9458 for PCa) and minimal Hausdorff Distance (HD < 0.6 mm), standardizing segmentation across clinicians and institutions. This eliminates subjective bias in PCa volume measurement and regional localization, providing a unified quantitative reference for PCa diagnosis and follow-up–essential for multicenter clinical studies and standardized management guidelines.

(2) Accurate PCa Volume and Boundary Assessment for Risk Stratification: PCa volume and extraprostatic extension (EPE) are core prognostic factors for PCa risk stratification (e.g., D'Amico classification) and treatment decision-making (active surveillance vs. radical prostatectomy vs. radiation therapy). Conventional methods often fail to segment small PCa foci ( ≤ 5 mm) and blurred boundaries, leading to inaccurate volume calculation and missed EPE. Our model's dual visual attention and multimodal fusion enable precise segmentation of irregular, small lesions, and minimal HD ensures accurate identification of PCa-prostate capsule boundaries. This allows reliable risk stratification, avoiding under-treatment of high-risk patients and over-treatment of low-risk patients.

(3) Reducing Radiologist Workload and Improving Diagnostic Efficiency: Clinical radiologists typically spend 15–20 min per patient on manual segmentation and PI-RADS grading. Our model achieves end-to-end automatic analysis in < 1 min per patient with high accuracy (PI-RADS ACC = 94.56%), serving as a clinical decision support system (CDSS). Radiologists can focus on reviewing high-risk cases (PI-RADS ≥4) instead of tedious manual work, reducing workload by over 80% and shortening diagnostic turnaround time for timely intervention.

(4) Promoting Biparametric MRI (bpMRI) Translational Application: bpMRI (T2WI+DWI) is a cost-effective alternative to mpMRI (17 vs. 45 min acquisition time, no contrast agent), but its clinical adoption is limited by the lack of accurate automatic analysis tools. Our model's superior multimodal fusion performance (DSC improvements of 2.12–12.39% over single-modal methods) validates bpMRI's clinical value, achieving diagnostic performance comparable to mpMRI with lower cost and no contrast-related adverse events. This promotes bpMRI adoption in resource-limited institutions and large-scale population screening, expanding access to high-quality PCa imaging diagnosis.

## 4 Conclusions

To solve the problems of morphological differences, variable sizes, blurred boundaries, and imbalanced categories in prostate and PCa regions on bpMRI images, and to improve the accuracy of automatic segmentation and PI-RADS automatic grading. This chapter proposes a multimodal and multitasking 3D prostate and PCa segmentation, as well as a PI-RADS automatic grading network. Design a dual visual attention module in the encoder stage, which consists of two layers of convolutional blocks. The first layer consists of standard convolutional blocks of different sizes, and the second layer consists of dilated convolutional blocks with different dilation rates. The collaboration between standard convolution and dilated convolution enables different features to be integrated into the dual visual attention module, resulting in visual features rich in detail information. We propose an SPM feature downsampling module. The design of SPM provides more detailed semantic information for the network, which is beneficial for information sharing between prostate and PCa. RLA layer was proposed at the decoder to obtain more precise global and local detail information. Design the HLFF module in the classification task stage, which is divided into two parts. The first part inputs the different features before and after the RLA layer. The second part of the input is a fusion feature map of two types of features, and then global average pooling and global maximum pooling are used to obtain global information. The fusion of the two parts structure obtains richer global and local feature information. A loss function has been designed to address the issue of class imbalance in prostate, PCa, and background areas. The prostate and PCa segmentation results of the proposed network on a large number of diverse male pelvic bpMRI datasets show that the segmentation results of the algorithm are highly consistent with the manual segmentation results of experts, and the prostate and PCa segmentation and classification also have higher segmentation performance compared to existing methods. The proposed multimodal multitasking network achieves state-of-the-art performance in prostate/PCa segmentation and PI-RADS grading, with significant advantages over recent SOTA multimodal transformer methods (2023-2025) in terms of core design philosophy, modality fusion strategy, attention mechanism, and task scope. The model's clinical task synergy design aligns with the actual workflow of PCa diagnosis, providing a one-stop clinical decision support system. External validation on the independent PROMISE12 dataset confirms its strong generalizability across different institutions and patient cohorts. The clinical implications of the model include standardizing segmentation results, improving risk stratification accuracy, reducing radiologist workload, and promoting the widespread adoption of bpMRI for PCa screening–especially in resource-limited regions. Future work will focus on optimizing the model's performance on smaller lesions and expanding the dataset to include more diverse patient populations and imaging protocols.

## Data Availability

The data analyzed in this study is subject to the following licenses/restrictions: This batch of datasets has not been made public yet. Requests to access these datasets should be directed to huangmx09@hainanu.edu.cn.
